# Supplementary material for: Assessing the accuracy of spectral indices obtained from Sentinel images using field research to estimate land degradation
Source: PLoS One. 2024 Jul 25;19(7):e0305758. doi: 10.1371/journal.pone.0305758 (PMC11271892; doi:10.1371/journal.pone.0305758)
Supplement: S1 Appendix — (DOCX) [file pone.0305758.s001.docx]

**The process of conducting field studies to determine the intensity of degradation in each plot**

The sub-criteria and criteria derived from Iranian Model Desertification Potential Assessment (IMDPA) procedures associated with the criteria and sub-factors that are consistent with the study area in each plot (Fig 1) are analyzed to achieve this goal. This process involves a scientific approach to identify the factors that contribute to land degradation and determine their intensity. The IMDPA criteria, which include soil, vegetation, wind erosion, and climate criteria, are used to assess the intensity of degradation. The selected indices for each criterion are assigned a score ranging from 1 to 2 based on the weight of each factor. The value of each criterion is then obtained as a geometric average of scores of single indices using a formula that involves the index of each criterion and the number of indices for each criterion.


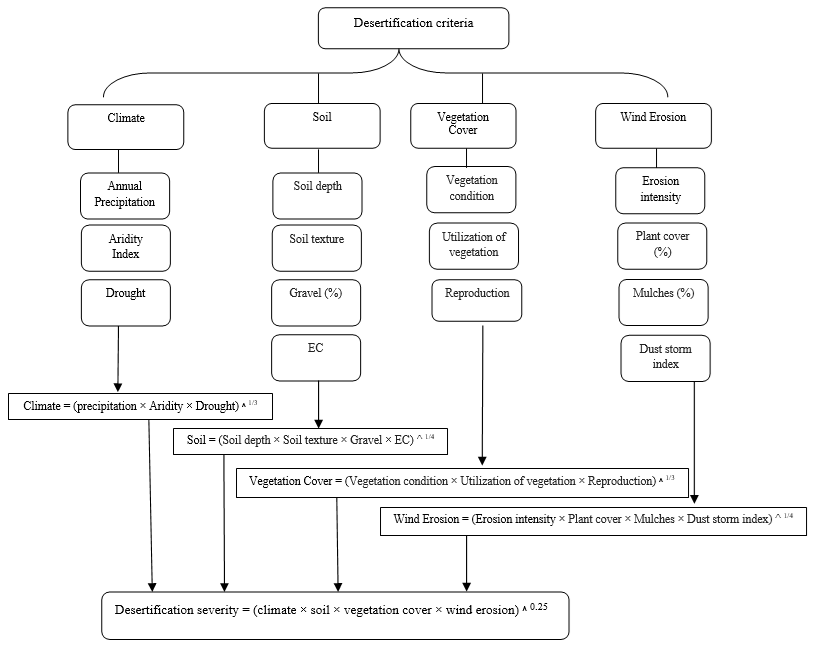


**Fig 1. Flowchart and different steps of determining the intensity of desertification based on the IMDPA model in each plot**

Determining the appropriate criteria and sub-factors for the evaluation of the land degradation process in each studied plot is shown in Table 1-4).

**Table 1. Indicators of Climate criterion**

| Range Of Score | Annual Precipitation (mm) | Aridity Index (WD^a^) | Drought Period (Year) |
| --- | --- | --- | --- |
| Low (1-1.24) | 280< | 150 - 180 | 3-4 |
| Medium (1.25-1.49) | 150 - 280 | 150 - 120 | 5-6 |
| High (1.5-1.74) | 75 - 150 | 120 - 90 | 6-7 |
| Very High (1.75-2) | <75 | 0 - 90 | >7 |

^a^: Wet Days

**Table 2. Indicators of Soil criterion**

| Range Of Score | Soil depth (cm) | Soil texture | Electrical conductivity(dsm^-1^) | Gravel (%) |
| --- | --- | --- | --- | --- |
| Low (1-1.24) | 80< | Clay, clay loam | <5 | <15 |
| Medium (1.25-1.49) | 50 - 80 | Fine loam | 5 - 8 | 15 - 35 |
| High (1.5-1.74) | 20 - 50 | Coarse loam | 9 -16 | 35 -75 |
| Very High (1.75-2) | <20 | Sand , sandy loam | >16 | >75 |

**Table 3. Indicators of Vegetation Cover criterion**

| Range Of Score | Vegetation condition | Utilization of vegetation cover | Reproduction |
| --- | --- | --- | --- |
| Low  (1-1.24) | Invader species are<5% of vegetation cover and annual plants >25%, Surface litter is >90%, Foliage cover of perennials is >85% | cutting of brush and uproot of the shrub is not seen, Stocking rate is equal to the rang capacity | Reproduction of plants are done naturally, The region does need not to reclamation projects, Decreases and increaser species are 70 and 30% respectively, and invaders ones are not seen |
| Medium  (1.25-1.49) | Invader species are5-20% of vegetation cover annual plants 25-50%, Surface litter is70-90%, Foliage cover of perennials is 15-30% | trees are more than annual biomass, Stocking rate is a little more than annual production | Reproduction of plants are access able with low expense, Range improvement projects are successfull and effective, Decreases and increaser species are dominant, and invaders ones are seen seldom |
| High  (1.5-1.74) | Invader species are20-50% of vegetation cover and annual plants are dominant, Surface litter is 30-70%, Foliage cover of perennials is 5-15% | cutting of brush, bush, and trees are apparent, Grazing is more than capacity | Regeneration of plants  involve high expense, Range improvement plans are a success to some extent, Invaders and increaser  species are dominant and decrease ones are not seen |
| Very High  (1.75-2) | Invader species are>50% of vegetation cover, and annual plants are dominant, Surface litter is <30%  foliage cover of perennials is <5% | Heavy cutting of brush, bush, and trees, Heavy stocking rate | Regeneration of plants are impossible (ecological problem), Range improvement projects have not successes till now, Invaders species are dominant, and increaser ones are not seen |

**Table 4. Indicators of Wind Erosion criterion**

| Range Of Score | Erosion intensity | Plant cover percentage | Mulches percent(gravel >2mm) | Dust storm index(days) |
| --- | --- | --- | --- | --- |
| Low (1-1.24) | 80< | PC>40 | MC>80 | <10 |
| Medium (1.25-1.49) | 50 - 80 | 20<PC<40 | 40<MC<80 | 10-30 |
| High (1.5-1.74) | 20 - 50 | 10<PC<20 | 20<MC<40 | 30-60 |
| Very High (1.75-2) | <20 | <10 | MC<20 | >60 |

The IMDPA criteria, which include soil, vegetation, wind erosion, and climate criteria, were used to assess the intensity of degradation. The selected indices for each criterion were assigned a score ranging from 1 to 2 based on the weight of each factor. The value of each criterion was then obtained as a geometric average of scores of single indices using a formula that involves the index of each criterion and the number of indices for each criterion.

Index- X Layer [(Layer−1). (Layer− 2) ...(Layer− n)] ^1/n^

where Index-X = given criteria;

Layer = index of each criterion;

N = number of indices for each criterion.

The degradation intensity was calculated as a geometric average of four criteria: soil, wind erosion, climate, and vegetation cover. The formula used to calculate degradation intensity is as follows:

degradation intensity = [ soil × wind erosion × climate × vegetation cover ] ^1/4^

Finally, the risk of degradation for each plot was classified into four subtypes according to Table 5.

| **Table 5. Classification of degradation intensity** | | | | |
| --- | --- | --- | --- | --- |
| order | 1 | 2 | 3 | 4 |
| Numerical value | 1 – 1.24 | 1.25 – 1.49 | 1.50 – 1.74 | 1.75 – 2 |
| Class | Low | Medium | High | Very High |

$$climate value=\left( precipitation \times drought index \times drought \right)^{\frac{\boldsymbol{1}}{\boldsymbol{3}}}$$

$$degradation intensity value=\left( climate value \times soil value \times wind erosion value \times vegetation cover value \right)^{\frac{1}{4}}$$
