# Supplementary material for: Assessing the accuracy of spectral indices obtained from Sentinel images using field research to estimate land degradation
Source: PLoS One. 2024 Jul 25;19(7):e0305758. doi: 10.1371/journal.pone.0305758 (PMC11271892; doi:10.1371/journal.pone.0305758)
Supplement: S1 Graphical abstract — (PDF) [file pone.0305758.s002.pdf]

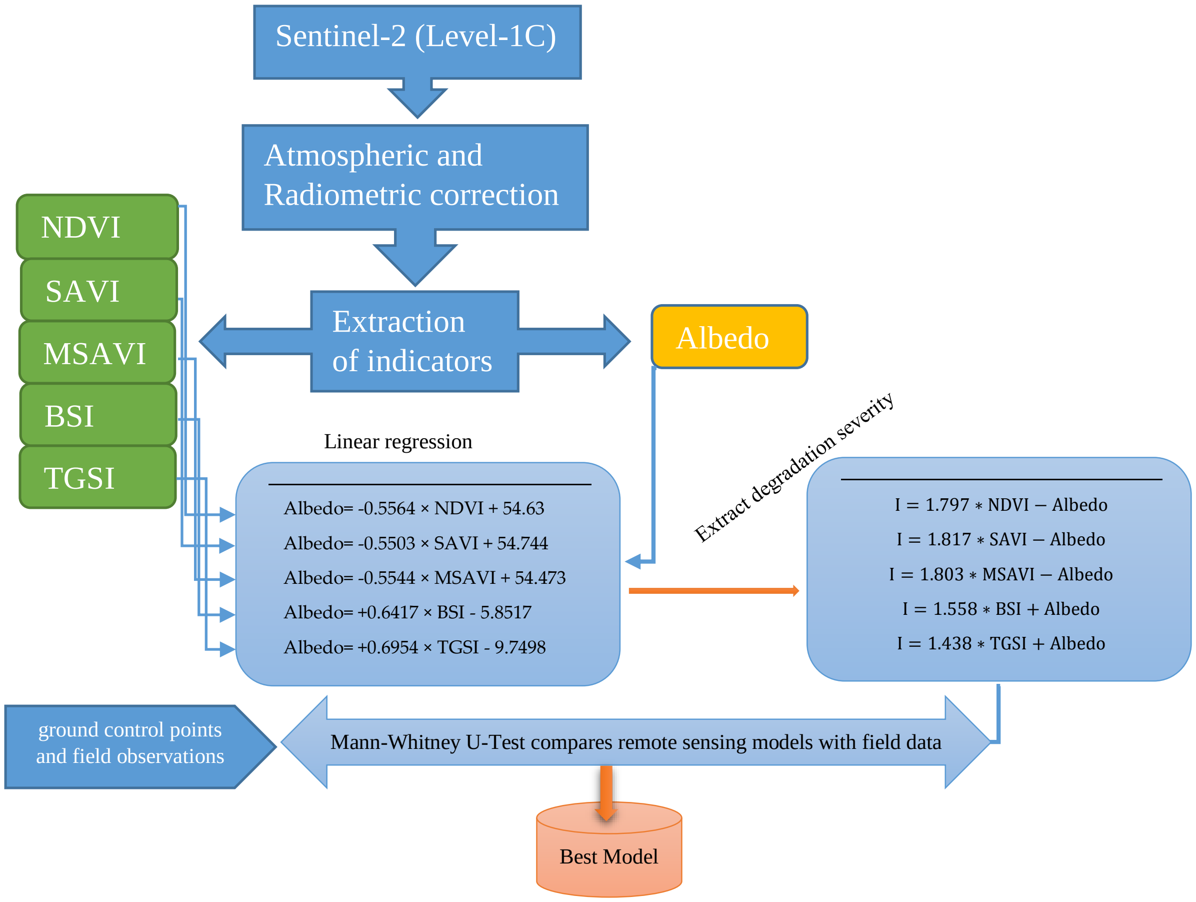

Graphical abstract of the methodology (Assessing the accuracy of spectral indices obtained from Sentinel images using field research to estimate land degradation)
