# Supplementary material for: Assessing the accuracy of spectral indices obtained from Sentinel images using field research to estimate land degradation
Source: PLoS One. 2024 Jul 25;19(7):e0305758. doi: 10.1371/journal.pone.0305758 (PMC11271892; doi:10.1371/journal.pone.0305758)
Supplement: Attachment — Submitted filename: PONE-D-23-27523_reviewer_t.pdf [file pone.0305758.s003.pdf]

# PLOS ONE

## Assessing the accuracy of spectral indices obtained from Sentinel images using field research to estimate land degradation

--Manuscript Draft--

| | |
|---|---|
| **Manuscript Number:** | PONE-D-23-27523 |
| **Article Type:** | Research Article |
| **Full Title:** | Assessing the accuracy of spectral indices obtained from Sentinel images using field research to estimate land degradation |
| **Short Title:** | Accuracy of Spectral Indices for Land Degradation Estimation |
| **Corresponding Author:** | farhad zolfaghari<br>Higher educational complex of saravan<br>saravan, IRAN, ISLAMIC REPUBLIC OF |
| **Keywords:** | Wind erosion; Soil degradation; Remote sensing; Baluchestan region; Degradation severity mapping; Sentinel-2 satellite; Albedo-spectral index correlation; Vegetation index |
| **Abstract:** | Wind erosion resulting from soil degradation is a significant problem in Iran's Baluchistan region. This study evaluated the accuracy of remote sensing models in assessing degradation severity through field studies. Sentinel-2 Multispectral Imager's (MSI) Level-1C satellite data was used to map Rutak's degradation severity in Saravan. The relationship between surface albedo and spectral indices (NDVI, SAVI, MSAVI, BSI, TGSI) was assessed. Linear regression establishes correlations between the albedo and each index, producing a degradation severity map categorized into five classes based on albedo and spectral indices. Accuracy was tested with 100 ground control points and field observations. The Mann-Whitney U-Test compares remote sensing models with field data. Results showed no significant difference ($P > 0.05$) between NDVI, SAVI, and MSAVI models with field data, while BSI and TGSI models exhibited significant differences ($P \leq 0.001$). The best model, BSI-NDVI, achieves a regression coefficient of 0.86. This study demonstrates the advantage of remote sensing technology for mapping and monitoring degraded areas, providing valuable insights into land degradation assessment in Baluchistan. By accurately identifying severity levels, informed interventions can be implemented to mitigate wind erosion and combat soil degradation in the region. |
| **Order of Authors:** | farhad zolfaghari |
| | Akhtar Ebrahimi |
| | Marzieh Ghodsi |
| | Fatemeh Narmashiri |
| **Additional Information:** | |
| **Question** | **Response** |
| **Financial Disclosure**<br><br>Enter a financial disclosure statement that describes the sources of funding for the work included in this submission. Review the submission guidelines for detailed requirements. View published research articles from *PLOS ONE* for specific examples.<br><br>This statement is required for submission and **will appear in the published article** if | The funders had no role in study design, data collection and analysis, decision to publish, or preparation of the manuscript. |

*Powered by Editorial Manager® and ProduXion Manager® from Aries Systems Corporation*

| | |
|---|---|
| the submission is accepted. Please make sure it is accurate.<br><br>**Unfunded studies**<br>Enter: *The author(s) received no specific funding for this work.*<br><br>**Funded studies**<br>Enter a statement with the following details:<br>• Initials of the authors who received each award<br>• Grant numbers awarded to each author<br>• The full name of each funder<br>• URL of each funder website<br>• Did the sponsors or funders play any role in the study design, data collection and analysis, decision to publish, or preparation of the manuscript?<br>• **NO** - Include this sentence at the end of your statement: *The funders had no role in study design, data collection and analysis, decision to publish, or preparation of the manuscript.*<br>• **YES** - Specify the role(s) played.<br><br>* typeset | |
| **Competing Interests**<br><br>Use the instructions below to enter a competing interest statement for this submission. On behalf of all authors, disclose any competing interests that could be perceived to bias this work—acknowledging all financial support and any other relevant financial or non-financial competing interests.<br><br>This statement is required for submission and **will appear in the published article** if the submission is accepted. Please make sure it is accurate and that any funding sources listed in your Funding Information later in the submission form are also declared in your Financial Disclosure statement.<br><br>View published research articles from *PLOS ONE* for specific examples. | The authors have declared that no competing interests exist. |

| | |
|---|---|
| **NO authors have competing interests**<br><br>Enter: *The authors have declared that no competing interests exist.*<br><br>**Authors with competing interests**<br><br>Enter competing interest details beginning with this statement:<br><br>*I have read the journal's policy and the authors of this manuscript have the following competing interests: [insert competing interests here]*<br><br>* typeset | |
| **Ethics Statement**<br><br>Enter an ethics statement for this submission. This statement is required if the study involved:<br><br>• Human participants<br>• Human specimens or tissue<br>• Vertebrate animals or cephalopods<br>• Vertebrate embryos or tissues<br>• Field research<br><br>Write "N/A" if the submission does not require an ethics statement.<br><br>General guidance is provided below. Consult the submission guidelines for detailed instructions. **Make sure that all information entered here is included in the Methods section of the manuscript.** | N/A |

| | |
|---|---|
| **Format for specific study types**<br><br>**Human Subject Research (involving human participants and/or tissue)**<br>• Give the name of the institutional review board or ethics committee that approved the study<br>• Include the approval number and/or a statement indicating approval of this research<br>• Indicate the form of consent obtained (written/oral) or the reason that consent was not obtained (e.g. the data were analyzed anonymously)<br><br>**Animal Research (involving vertebrate animals, embryos or tissues)**<br>• Provide the name of the Institutional Animal Care and Use Committee (IACUC) or other relevant ethics board that reviewed the study protocol, and indicate whether they approved this research or granted a formal waiver of ethical approval<br>• Include an approval number if one was obtained<br>• If the study involved *non-human primates*, add *additional details* about animal welfare and steps taken to ameliorate suffering<br>• If anesthesia, euthanasia, or any kind of animal sacrifice is part of the study, include briefly which substances and/or methods were applied<br><br>**Field Research**<br><br>Include the following details if this study involves the collection of plant, animal, or other materials from a natural setting:<br>• Field permit number<br>• Name of the institution or relevant body that granted permission | |
| **Data Availability**<br><br>Authors are required to make all data underlying the findings described fully available, without restriction, and from the time of publication. PLOS allows rare exceptions to address legal and ethical concerns. See the PLOS Data Policy and FAQ for detailed information. | Yes - all data are fully available without restriction |

*Powered by Editorial Manager® and ProduXion Manager® from Aries Systems Corporation*

| | |
|---|---|
| A Data Availability Statement describing where the data can be found is required at submission. Your answers to this question constitute the Data Availability Statement and **will be published in the article**, if accepted.<br><br>**Important:** Stating 'data available on request from the author' is not sufficient. If your data are only available upon request, select 'No' for the first question and explain your exceptional situation in the text box.<br><br>Do the authors confirm that all data underlying the findings described in their manuscript are fully available without restriction? | |
| **Describe where the data may be found in full sentences. If you are copying our sample text, replace any instances of XXX with the appropriate details.**<br><br>• If the data are **held or will be held in a public repository**, include URLs, accession numbers or DOIs. If this information will only be available after acceptance, indicate this by ticking the box below. For example: *All XXX files are available from the XXX database (accession number(s) XXX, XXX.*).<br>• If the data are all contained **within the manuscript and/or Supporting Information files**, enter the following: *All relevant data are within the manuscript and its Supporting Information files.*<br>• If neither of these applies but you are able to provide **details of access elsewhere**, with or without limitations, please do so. For example:<br><br>*Data cannot be shared publicly because of [XXX]. Data are available from the XXX Institutional Data Access / Ethics Committee (contact via XXX) for researchers who meet the criteria for access to confidential data.*<br><br>*The data underlying the results presented in the study are available from (include the name of the third party* | All relevant data are within the manuscript and its Supporting Information files. |

| | |
|---|---|
| *and contact information or URL).*<br>• This text is appropriate if the data are owned by a third party and authors do not have permission to share the data.<br><br>* typeset | |
| Additional data availability information: | |

Manuscript

Click here to access/download;Manuscript;Spectral Indices for Land Degradation Estimation.docx

# Assessing the accuracy of spectral indices obtained from Sentinel images using field research to estimate land degradation

**Head running: Accuracy of Spectral Indices for Land Degradation Estimation**

Akhtar Ebrahimi[1], Farhad. Zolfaghari[2*], Marzieh Ghodsi[3], Fatemeh Narmashiri[2]

1. Msc student of Higher Education Complex of Saravan, Iran, akhtarebrahimi1370@gmail.com

2. Assistant Professor, Higher education complex of Saravan, Iran, Fateme.narmashiri@gmail.com
3. Ph.D. Physical Geography(Geomorphology), Faculty of Geography, University of Tehran, mghodsi@gmail.com
* Correspondence: Zol.farhad@gmail.com; +989153424792

Abstract: Wind erosion resulting from soil degradation is a significant problem in Iran's Baluchistan region. This study evaluated the accuracy of remote sensing models in assessing degradation severity through field studies. Sentinel-2 Multispectral Imager's (MSI) Level-1C satellite data was used to map Rutak's degradation severity in Saravan. The relationship between surface albedo and spectral indices (NDVI, SAVI, MSAVI, BSI, TGSI) was assessed. Linear regression establishes correlations between the albedo and each index, producing a degradation severity map categorized into five classes based on albedo and spectral indices. Accuracy was tested with 100 ground control points and field observations. The Mann-Whitney U-Test compares remote sensing models with field data. Results showed no significant difference ($P > 0.05$) between NDVI, SAVI, and MSAVI models with field data, while BSI and TGSI models exhibited significant differences ($P \leq 0.001$). The best model, BSI-NDVI, achieves a regression coefficient of 0.86. This study demonstrates the advantage of remote sensing technology for mapping and monitoring degraded areas, providing valuable insights into land degradation assessment in Baluchistan. By accurately identifying severity levels, informed interventions can be implemented to mitigate wind erosion and combat soil degradation in the region.

**Keywords:** Wind erosion; Soil degradation; Remote sensing; Baluchestan region; Degradation severity mapping; Sentinel-2 satellite; Albedo-spectral index correlation; Vegetation index

## 1. Introduction

Land degradation is a significant environmental issue in dry areas, leading to soil productivity loss, vegetation decline, and increased desertification. Studying soil and vegetation changes is crucial for understanding these processes' dynamics and developing effective land management and conservation strategies. Reflectance from the soil and vegetation surface plays a significant role in monitoring environmental changes (Gbetkom et al., 2018). Vegetation stabilizes dry areas, reducing erosion effects and determining the intensity of desertification (Querino et al.,

2016). Reduction in vegetation cover and biomass directly contributes to land degradation, increasing surface albedo (Ma et al., 2011).

Various factors contribute to land degradation in dry areas, including ecosystem changes, landslides, deforestation, human impact, and soil erosion (Chikhaoui et al., 2005). In degraded areas with thin vegetation, albedo increases, indicating higher surface reflectance (Cordeiro et al., 2015). The decrease in vegetation cover reduces humidity, further increasing surface albedo in these regions (Pan & Li, 2013). Monitoring changes in albedo and vegetation indices can help identify and assess the extent of land degradation (Zolfaghari & Abdollahi, 2022).

Remote sensing technology has proven valuable in mapping and monitoring desertification intensity in arid and semi-arid regions. Previous studies have utilized remote sensing data, such as Landsat OLI satellite imagery, to develop classification algorithms and spectral indices for assessing desertification (Afrasinei et al., 2017, 2018; Lamchin et al., 2016). In 2011, researchers proposed a model based on the albedo index and normalized vegetation difference index, effectively reflecting the desertification of the Earth's surface and changes in desertified areas (Ma et al., 2011).

Spectral composition analysis has been employed to study vegetation, water, and bare soil using satellite imagery, reducing data noise through techniques like Minimum Noise Fraction (MNF) and Pixel Purity Index (PPI) (Pan & Li, 2013). The state and degree of desertification can be determined by analyzing the spectral-temporal characteristics of vegetation and albedo obtained from Landsat satellite images (Karnieli et al., 2014). Spatio-temporal patterns of desertification have been explored using indicators such as the Modified Soil-Adjusted Vegetation Index (MSAVI), Fractional Vegetation Cover (FVC), and Temperature–Vegetation Drought Index (TVDI), alongside surface temperature and albedo (Han et al., 2015).

The status of the land surface regarding vegetation biomass, landscape pattern, and climatology at a small scale has been investigated and evaluated using the NDVI, TGSI, and Albedo indices

(Lamchin et al., 2016). The researchers did not observe any correlation between the NDVI and Albedo indices or between the NDVI and TGSI. However, they found a significant correlation (0.77 to 0.92) between the Albedo and TGSI indices in non-desertification areas. In this study, they demonstrated that based on these indices, the region's desertification is increasing. They also showed that by identifying changes in the levels of NDVI, TGSI, and surface Albedo, the process of desertification could be identified, and the highest TGSI values were observed in areas experiencing severe desertification.

Researchers have employed the extraction of NDVI, BSI, and Albedo indices from Landsat satellite images and the use of the CVA model to investigate the determinants of the direction and magnitude of these indices and evaluate the degradation or improvement of land conditions over different periods (Bercerril-Pina et al., 2016). This study demonstrated the relationship between NDVI and Albedo as an indicator of desertification. Additionally, other researchers focused on studying soil degradation using the Albedo and MSAVI indices, showcasing the intensity of soil degradation through remote sensing technology (Feng et al., 2018). The examination and study of a regional geographical model, along with the investigation of three indices (NDVI, TGSI, MSAVI), and surface Albedo revealed that the relationship between the Albedo and MSAVI indices is more suitable for obtaining a more robust and appropriate desertification intensity map compared to the other two indices (Wei et al., 2018). The assessment of desertification intensity based on the spatial characteristics of Albedo and NDVI, as well as Albedo and MSAVI, showed that a decrease in vegetation coverage in the studied areas led to a decrease in the values of NDVI and MSAVI while increasing the Albedo values (Li et al., 2000; Wang et al., 2017).

Various methods have been used in the Sistan and Baluchestan provinces to evaluate and classify desertification intensity. However, challenges such as high costs, regional size, and limited access due to security issues hinder comprehensive assessments (Mohamad Ghasemi et

al., 2008; Parvari Asl et al., 2010; Fozuni et al., 2012; Eftekhari et al., 2015; Zolfaghari & Khosravi, 2016).

The use of multispectral satellite data, such as Sentinel-2, for identifying compacted soil has been shown to be effective (Mzid et al., 2021). Furthermore, the results of studying two indices, NDVI and BSI, using Sentinel-2 images indicated that, with a 95% confidence level, vegetated areas and bare soil areas could be distinguished using these two indices (Mzid et al., 2021). The BSI index has been widely utilized for identifying areas with bare soil and no vegetation cover (Polykreties et al., 2020).

The present study aims to evaluate the performance of spectral indices obtained from remote sensing and field studies in the Rutak Saravan region to enhance the accuracy of land degradation assessment. Sentinel satellite data will be utilized to investigate the relationship between different spectral indices and albedo. The study aims to identify the most reliable indicator with the minimal error by comparing the results with field data. This research will contribute valuable insights for monitoring and managing land degradation in the region, aiding in sustainable land management and environmental conservation efforts.

## 2. Materials and Methods

### *2.1 Study Area*

The study area, encompassing 46,656.5 hectares, is situated within the latitude range of 28° 00′ to 28° 16′ North and the longitude range of 62° 29′ to 62° 47′ East (Fig 1). The Rutak-Saravan region is located approximately 150 km away from the center of Saravan City in Sistan and Baluchistan province, Iran. The elevation in the region ranges from 530 meters in the western part to 480 meters in the eastern part, near the border of Pakistan. Geomorphologically, the region belongs to the Pediments unit, which extends towards the Mashkel playa in Pakistan, as determined by the slope index.

The region experiences a Mediterranean rainfall regime, with a peak winter precipitation of 51.15 mm and an average long-term precipitation of 35.11 mm. The annual average temperature is 22.1 °C. The hottest month is July, with an average temperature of 39.7 °C, while the coldest month is January, with an average temperature of 16.7 °C (Zolfaghari & Khosravi, 2016).

**Fig 1.** Location of the study area

*2.2 Softwares*

The Sentinel Application Platform (SNAP) software was used to preprocess and process Sentinel satellite images. Additionally, statistical analysis and regression were conducted using $SPSS_{25}$ software. The degradation severity map was generated as an output using $ArcGIS_{10.6}$ software. These software tools were chosen for their established reliability and functionality in remote sensing data analysis and geographic information system (GIS) mapping. Using SNAP allowed for efficient handling and manipulation of the Sentinel satellite images. $SPSS_{25}$ facilitated the statistical analysis and regression modeling necessary for evaluating the relationship between spectral indices and albedo. $ArcGIS_{10.6}$, on the other hand, provided a robust platform for spatial analysis and the creation of the degradation severity map. The study aimed to ensure accurate and reliable results in assessing land degradation in the Rutak-Saravan region by employing these software applications.

*2.3 Remote Sensing Dataset and Preprocessing*

The initial phase involved the preparation of satellite images and the execution of image processing operations. Sentinel-2A data captured by the MSI-L1C sensor on August 2, 2020, was selected to accomplish this. The required Sentinel data was obtained from the Open Access Hub of the European Space Agency, accessible at https://scihub.copernicus.eu/dhus/#/home. Subsequently, the SNAP software was utilized to preprocess and process the Sentinel satellite images. Vegetation indices, including NDVI, SAVI, MSAVI, BSI, TGSI, and Albedo, were computed using Equations (1) - (6), as described by Rahimi et al. (2014).

$$\mathrm{NDVI} = \frac{\mathrm{NIR-RED}}{\mathrm{NIR+RED}} \quad (1)$$

$$\mathrm{MSAVI} = \frac{\mathrm{NIR-RED}*(1+\mathrm{L})^2}{\mathrm{NIR+RED+L}} \quad (2)$$

$$\mathrm{SAVI} = \frac{(1+\mathrm{L})\times(\mathrm{NIR-RED})}{\mathrm{NIR+RED+L}} \quad (3)$$

$$\mathrm{TGSI} = \frac{(\mathrm{R-B})}{(\mathrm{R+B+G})} \quad (4)$$

$$\mathrm{BSI}=\frac{(\mathrm{SWIR+R})-(\mathrm{NIR+B})}{(\mathrm{SWIR+R})+(\mathrm{NIR+B})}\times 100 + 100 \quad (5)$$

In these equations, RED is the red band or number 4, and NIR is the near-infrared band or number 8 of the Sentinel 2 satellite. The values of the coefficients in relations 1 to 5 were used as follows:

$\alpha = 0 \cdot 96916$
$b = 0 \cdot 84726$
$L = 0 \cdot 5$

*2.4 Surface Albedo*

The surface albedo index was calculated using Liang's relation, tailored explicitly for Sentinel-2 sensor images. This relation, described by Naegeli et al. (2017), was employed to determine the albedo values according to the Equation (6):

$$\alpha = 0 \cdot 356b_2 + 0 \cdot 130b_4 + 0 \cdot 373b_8 + 0 \cdot 085b_{11} + 0 \cdot 072b_{12} - 0 \cdot 0018 \quad (6)$$

In this equation, α represents the surface albedo value, and the symbol refers to the band number of the Sentinel-2 optical satellite.

A crucial step was to convert the bands with resolutions of 10 and 20 meters to a consistent spatial resolution of 10 meters to calculate the albedo. This conversion was achieved by utilizing the resampling command in the SNAP software. Accurate and consistent albedo values could be derived by harmonizing the resolution across all bands for further analysis and interpretation.

The investigation focused on the August summer season to minimize errors resulting from surface moisture and the absence of annual species. This period was chosen because the soil surface is typically dry, and only perennial species are present, which play a crucial role in soil stability and protection.

*2.5 Land degradation severity map*

To establish the correlation between vegetation indices (NDVI, SAVI, TGSI, MSAVI, BSI) and Surface Albedo, we employed a linear regression model using 520 randomly selected pixels. We calculate the slope coefficient of the regression line for each vegetation index and albedo to generate the Land degradation severity map by Equation (7).

$$\mathrm{DI} = \mathrm{a} * \mathrm{Index} - \mathrm{Albedo} \qquad (7)$$

In this equation, “DI” stands for degradation intensity, and "a” stands for the coefficient value resulting from the regression between the albedo index and the corresponding spectral index, which is determined by dividing one by the coefficient of the vegetation index (Bercerril-Pina et al., 2016) and “Index” is the examined spectral indices.

The Jenks natural breaks classification method (Jenks, 1967) was employed to classify the data into five degrees of land degradation, namely Null, Low, Medium, High, and Very High (Han et al., 2015; Bercerril-Pina et al., 2016; Yaduvanshi et al., 2015). To assess the accuracy of the models based on spectral indices, 100 plots measuring 10 x 10 meters were randomly selected across different parts of the study area, aligning with the pixel size of the Sentinel-2 images. These plots represented varying degrees of land degradation and were sampled during the same season as the time of the Sentinel image, specifically August 2020.

*2.6 Correlation Analysis*

The Mann-Whitney U-Test was employed to analyze the relationship between each remote sensing model and field observations. Additionally, the Root Mean Square Error (RMSE) was used as a quantitative index to evaluate the performance of the models using both remote

sensing and field data. The RMSE indicates the magnitude of error in the models, with a lower value indicating better performance. The calculation of RMSE is based on the Equation (8):

$$\mathrm{RMSE} = \sqrt{\frac{\sum_{K=0}^{K}(X_k - Y_k)^2}{K}} \quad (8)$$

$X_K$: observed data in field research

$Y_k$: values obtained from the model

K: number of data

## 3. Results

### *3.1 Relationship Between Albedo and Spectral Indices*

The relationship between the NDVI and Albedo indices was investigated through linear regression analysis, revealing a significant negative correlation between these two indices (Fig 2A). The correlation coefficient was determined to be 0.825 at a 99% confidence level. It was observed that as the NDVI index increased, the surface albedo exhibited a decrease.

The results of the linear regression analysis between the SAVI and Albedo indices demonstrated a strong negative correlation with a correlation coefficient of 0.799 at a 99% confidence level (Fig 2B). Similarly, the MSAVI and Albedo indices exhibited a negative correlation with a correlation coefficient of 0.790 (99% confidence level) (Fig 2C). Furthermore, the linear regression analysis between the BSI and Albedo indices revealed a positive correlation coefficient of 0.763 (99% confidence level) (Fig 2D), indicating that an increase in the BSI index corresponds to an increase in the Albedo index.

The relationship between the TGSI and Albedo indices was positively correlated, with a correlation coefficient of 0.836 (99% confidence level) (Fig 2E). The linear regression analysis between the BSI and NDVI indices showed a strong negative correlation with a correlation coefficient of -0.927 (99% confidence level) (Fig 2F). Similarly, the TGSI and NDVI indices exhibited a negative correlation with a correlation coefficient of -0.897 (99% confidence level) (Figure 2G).

Overall, the examination of the relations between the Albedo index and each of the investigated vegetation indices (NDVI, SAVI, MSAVI, BSI, and TGSI) revealed that the highest correlation was observed between the TGSI and Albedo indices with a correlation coefficient of +0.836. Conversely, the lowest correlation was observed between the BSI index and albedo, with a correlation coefficient of +0.763 in the studied area.

Furthermore, the analysis of the relationship between NDVI, BSI, and TGSI indicated that the strongest negative correlation existed between NDVI and BSI, with a correlation coefficient of 0.927. Table 1 displays the relationship between surface albedo and each studied index and the correlation between NDVI and BSI/TGSI indices in the studied area.

**Fig 2.** Linear regression relationship between albedo and other indicators; A: Albedo-NDVI, B: Albedo-SAVI, C; Albedo-MSAVI, D: Albedo-BSI, E: Albedo-TGSI, F: NDVI-BSI, and G: NDVI-TGSI

**Table 1**. The results of the linear regression relationship between the albedo and spectral indices

| Variables | Regression equation | Regression coefficient | Std. error | P value |
|---|---|---|---|---|
| NDVI-Albedo | Albedo= -0.5564 × NDVI + 54.63 | 0.6813 | -0.825 | 0.0001 |
| SAVI-Albedo | Albedo= -0.5503 × SAVI + 54.744 | 0.6396 | -0.799 | 0.0001 |
| MSAVI-Albedo | Albedo= -0.5544 × MSAVI + 54.473 | 0.6244 | -0.790 | 0.0001 |
| BSI-Albedo | Albedo= +0.6417 × BSI - 5.8517 | 0.5834 | +0.763 | 0.0001 |
| TGSI-Albedo | Albedo= +0.6954 × TGSI - 9.7498 | 0.6999 | +0.836 | 0.0001 |
| BSI-NDVI | NDVI= -0.7443 × BSI + 90.439 | 0.8604 | -0.927 | 0.0001 |
| TGSI-NDVI | NDVI= -0.728 × TGSI + 90.343 | 0.8057 | -0.897 | 0.0001 |

## *3.2. Land Degradation Severity Map*

The severity of land degradation was assessed using the coefficients derived from the linear regression analysis between the Albedo index and the vegetation indices, as presented in Table 1. These coefficients were then utilized to generate the land degradation severity equation, as illustrated in Table 2.

**Table 2.** Land degradation severity equation.

| Indices | Equation |
|---|---|
| NDVI-Albedo | $\mathrm{I} = 1.797 * \mathrm{NDVI} - \mathrm{Albedo}$ |
| SAVI-Albedo | $\mathrm{I} = 1.817 * \mathrm{SAVI} - \mathrm{Albedo}$ |
| MSAVI-Albedo | $\mathrm{I} = 1.803 * \mathrm{MSAVI} - \mathrm{Albedo}$ |
| BSI-Albedo | $\mathrm{I} = 1.558 * \mathrm{BSI} + \mathrm{Albedo}$ |
| TGSI-Albedo | $\mathrm{I} = 1.438 * \mathrm{TGSI} + \mathrm{Albedo}$ |
| BSI-NDVI | $\mathrm{I} = 1.343 * \mathrm{BSI} - \mathrm{NDVI}$ |
| TGSI-NDVI | $\mathrm{I} = 1.373 * \mathrm{TGSI} - \mathrm{NDVI}$ |

The results of the land degradation severity classification based on the Albedo-NDVI model revealed that the majority of the studied area, comprising 44.32%, falls into the very high degradation class. In contrast, less than one percent (0.36) of the area is classified as having no degradation (Fig 3A). Furthermore, classification based on the Albedo-SAVI model demonstrated that the highest level of degradation (43.11% of the area) was observed in the very high degradation class, while the class representing areas without degradation accounted for only 0.45% of the region (Fig 3B).

Analyzing the severity of degradation using the Albedo-MSAVI model demonstrated that the highest level of degradation, at 41.78%, was observed in the high class. In contrast, the lowest level, at 0.46%, was found in the without degradation class (Fig 3C). Similarly, the Albedo-BSI model indicated that the highest level of degradation, with 37.85%, occurred in the high class. In contrast, the lowest level, at 0.56%, was observed in the without degradation class (Fig 3D). Regarding the Albedo-TGSI model, the analysis revealed that the very high class represented the medium level of degradation, covering 32.78% of the area. In contrast, the null degradation class accounted for the lowest level, encompassing only 5.38% of the region (Fig 3E).

**Fig 3.** Land degradation severity map based on albedo index and spectral indices. A: Albedo-NDVI, B: Albedo-SAVI, C; Albedo-MSAVI, D: Albedo-BSI, and E: Albedo-TGSI,

According to the NDVI-BSI model, the severity of degradation in the area was observed in the high degradation class, which covers 37.81% of the total area. In contrast, the null degradation

class, which represents the lowest level of degradation, only covers 0.58% of the area (Fig. 3F). Meanwhile, the NDVI-TGSI model revealed that the very high degradation class had the highest level of degradation in the area, covering 48.01% of the total area. Conversely, the null degradation class accounted for only 0.25% of the total area, representing the lowest level of degradation (Fig. 3G).

**Fig 4. The map of land degradation severity based on the NDVI, BSI, and TGSI indices examined respectively: F: NDVI-BSI, and G: NDVI-TGSI**

The percentage of land degradation based on each model is summarized separately in Table 4.

**Table 4.** Percentage of land degradation severity classes based on the (I=a*Index-Albedo) model

| **Severity degradation Class** | | | **Percentage of Degradation Severity** | | | | | |
|---|---|---|---|---|---|---|---|---|
| | IMDPA | NDVI-TGSI | NDVI-BSI | TGSI | BSI | MSAVI | SAVI | NDVI |
| Very high | 30.00 | 48.01 | 21.32 | 11.40 | 23.06 | 39.72 | 43.11 | 44.32 |
| High | 40.00 | 39.21 | 37.81 | 31.45 | 37.85 | 41.78 | 38.38 | 38.78 |
| Medium | 19.00 | 10.11 | 30.02 | 32.78 | 28.97 | 13.93 | 14.00 | 13.13 |
| Low | 6.00 | 2.43 | 10.26 | 18.99 | 9.55 | 4.11 | 4.06 | 3.42 |
| Null | 5.00 | 0.25 | 0.58 | 5.38 | 0.56 | 0.46 | 0.45 | 0.36 |

### *3.3 Mann-Whitney Test Results*

The Mann-Whitney test was conducted to assess the severity of land degradation using the Albedo index and various spectral indices derived from remote sensing and field data. The results revealed that in the studied area, the severity of degradation based on the NDVI, SAVI, and MSAVI indices did not exhibit a significant difference compared to the field data at a 95% confidence level (α=0.05), with p-values greater than 0.05. However, the BSI and TGSI indices showed a significant difference with the field data, as indicated by p-values of 0.00. Furthermore, the Mann-Whitney test demonstrated that the BSI-NDVI model did not significantly differ from the field data (p-value=0.006). In contrast, the TGSI-NDVI model showed a significant difference with a p-value of 0.336.

**Table 5.** Mann-Whitney test results between the model obtained from spectral indices and field data

| Index | group | N | Mean Rank | Sum of Ranks | Mann-Whitney U | Wilcoxon W | Z | Asymp. Sig (2-tailed) |
|---|---|---|---|---|---|---|---|---|
| NDVI-Albedo | 1 | 100 | 107.40 | 10739.50 | 4310.500 | 9360.500 | -1.777 | 0.076 |
| | 2 | 100 | 93.61 | 9360.50 | | | | |
| SAVI-Albedo | 1 | 100 | 106.70 | 10669.50 | 4380.500 | 9430.500 | -1.597 | 0.110 |
| | 2 | 100 | 94.31 | 9430.50 | | | | |
| MSAVI-Albedo | 1 | 100 | 105.65 | 10564.50 | 4485.500 | 9535.500 | -1.326 | 0.185 |
| | 2 | 100 | 95.36 | 9535.50 | | | | |
| BSI-Albedo | 1 | 100 | 72.17 | 7217.00 | 2167.000 | 7217.000 | -7.096 | 0.000 |
| | 2 | 100 | 128.83 | 12883.00 | | | | |
| TGSI-Albedo | 1 | 100 | 80.55 | 8054.50 | 3004.500 | 8054.500 | -5.017 | 0.000 |
| | 2 | 100 | 120.46 | 12045.50 | | | | |
| BSI-NDVI | 1 | 100 | 89.81 | 8980.50 | 3930.500 | 8980.500 | -2.742 | 0.006 |
| | 2 | 100 | 111.20 | 11119.50 | | | | |
| TGSI-NDVI | 1 | 100 | 104.21 | 10420.50 | 4629.500 | 9679.500 | -0.962 | 0.336 |
| | 2 | 100 | 96.80 | 9679.50 | | | | |

### *3.4 Model Evaluation and Comparison with Field Data Based on RMSE*

The comparison of RMSE values between the Albedo models, spectral indices, and field-observed data revealed that in the studied area, the MSAVI-Albedo indices exhibited the highest consistency with the field survey data (RMSE=0.65), as shown in Table (6). An RMSE value of zero or lower indicates a perfect fit for the data. Therefore, the severity of degradation results obtained from the NDVI-TGSI model (RMSE=0.66) outperformed other models in terms of accuracy.

**Table 6.** Quantitative RMSE Index Comparison Between Spectral Index Models and Field Data

| | NDVI-Albedo | SAVI-Albedo | MSAVI-Albedo | BSI-Albedo | TGSI-Albedo | BSI-NDVI | TGSI-NDVI |
|---|---|---|---|---|---|---|---|
| RMSE | 0.692 | 0.678 | 0.655 | 2.204 | 2.184 | 1.153 | 0.663 |

## 4. Discussion

The objective of this study was to identify the best model for estimating the intensity of land degradation by comparing remote sensing indices using Sentinel satellite data and field data.

The Sentinel satellite images with a 10-meter resolution were used as the basis for creating vegetation and soil cover indices. Seven different combinations of these indices (Albedo-NDVI, Albedo-SAVI, Albedo-MSAVI, Albedo-BSI, Albedo-TGSI, NDVI-BSI, and NDVI-TGSI) were evaluated to assess their impact on identifying degraded areas. The goal was to determine the best combination based on the accuracy of the results obtained through field surveys.

The results of the investigation into the relationship between the Albedo index and vegetation cover indices (NDVI, SAVI, MSAVI, BSI, and TGSI) demonstrated that the highest correlation in the study area was observed between the TGSI-Albedo index, with a value of +0.836. In contrast, the lowest correlation was found between the BSI-Albedo index, with a value of +0.763.

Furthermore, the analysis of the relationship between the NDVI index and the BSI and TGSI indices indicated that the strongest negative correlation existed between the NDVI and BSI indices, with a correlation coefficient of +0.927.

The results of the Mann-Whitney U test examining the intensity of land degradation based on the Albedo index, spectral indices, and field data indicated that in the study area, with 95% confidence ($\alpha$=0.05), the models of land degradation intensity derived from the NDVI, SAVI, and MSAVI indices did not show a significant difference compared to the models derived from field measurements.

Researchers conducted a study in a part of Mongolia, China, investigating the relationship between three indices, NDVI, TGSI, MSAVI, and Albedo, to generate a desertification intensity map (Wei, et al. 2020). The results of their study differed from the findings of the present research. They demonstrated that the relationship between the Albedo-MSAVI indices compared to the NDVI and TGSI indices with albedo is more robust and more suitable for determining the desertification intensity map. This difference might be attributed to using

satellite images with higher spatial accuracy (Sentinel-2 images with a 10-meter resolution) compared to Landsat images with a 30-meter resolution.

It is a scientific fact that the amount of reflected light from the Earth's surface in the range of 0.2 to 0.6 micrometers increases due to a decrease in vegetation cover (Zhao et al., 2018).

The results of the linear regression analysis between the Albedo index and spectral indices (BSI, MSAVI, SAVI, NDVI, and TGSI) in the Rutak region of Sistan and Baluchestan province indicate a negative relationship (correlation coefficient Albedo-NDVI = -0.82), which is consistent with the findings of other researchers in dry areas (Pan & Li, 2013).

Studies using the Change Vector Analysis (CVA) technique have confirmed using the NDVI and Albedo indices to determine degradation intensity in different periods (Karnieli et al., 2014).

Other studies based on the albedo and vegetation cover indices (Bercerril-Pina et al., 2016) have also shown a strong correlation between the NDVI and Albedo indices, consistent with the present research findings.

Examining various vegetation cover indices and albedo results indicates that an increase in the MSAVI, SAVI, and NDVI indices leads to a decrease in the surface albedo. Areas with high albedo indicate vegetation degradation and soil exposure. In the studied area, in regions classified as having high levels of degradation, the surface Albedo is high, while the vegetation cover index shows a lower value. These findings are consistent with those of studies conducted in other parts of the world (Ma et al., 2011).

The correlation analysis between the BSI and TGSI indices with albedo indicates a positive relationship. However, the intensity map of degradation obtained from these indices shows a significant difference from the field data, as confirmed by the Mann-Whitney U test with a significance level of 0.00. Therefore, these two indices cannot be used to monitor this area's degradation.

The results of the examination of the Root Mean Square Error (RMSE) between the models obtained from the Albedo index and each of the spectral indices derived from remote sensing and field data also showed that the intensity of degradation derived from the MSAVI-Albedo index has the highest proximity to the field data with an RMSE of 0.65. This indicates a closer agreement than the other indices, NDVI and SAVI.

Furthermore, the intensity of degradation obtained from the NDVI-TGSI model with an RMSE of 0.66 is higher than other models, indicating that the degradation intensity map derived from these two indices has higher accuracy compared to vegetation cover indices with Albedo (Hooshyar et al., 2012).

The classification of degradation intensity in the Rutak Saravan area based on the Albedo-MSAVI model, which had less error with the field data, indicates that 46.4% of the area is not classified as degraded, 11.4% falls into the low degradation class, 13.93% is classified as moderate degradation, 41.78% is classified as severe degradation, and 39.72% of the area is classified as very severe degradation. These are inconsistent with studies conducted in the study area using other standard models to map desertification intensity, which identified the loss of vegetation cover as one of the main causes of degradation and desertification (Parvari Asl et al., 2010; Zolfaghari & Khosravi, 2016).

The results of determining desertification intensity based on the IMDPA model in the study area (Zolfaghari & Khosravi, 2016) showed that one of the critical factors is the loss of vegetation cover. It can be concluded that the best spectral indices with the lowest RMSE for mapping degradation intensity, which also have the highest correlation with the Albedo index, are MSAVI, TGSI-NDVI, SAVI, and NDVI. Therefore, it can be said that the results of classifying the degradation intensity of the study area in the present research based on the Albedo model and each of the investigated indices, especially MSAVI, are in a favorable position for identifying degraded areas and determining degradation intensity classes. This

finding is consistent with other studies in this field (Han et al., 2015; Yaduvanshi et al., 2015; Bercerril-Pina et al., 2016, and others).

## 5. Conclusions

One of the objectives of studying various indices for determining the intensity of degradation in different areas is to achieve the highest accuracy in using the desired index. In this study, the relationship between different spectral indices and albedo was evaluated using remote sensing technology and linear regression.

This method effectively identifies breakpoints between classifications with the optimized JENKS algorithm based on the nature of the data and their inherent grouping. In the present study, the degradation intensity in the dry region of Balochistan, Iran, was extracted using remote sensing techniques and based on multispectral Sentinel-2 images. The degradation intensity was examined based on the spectral reflectance from the ground surface and the spatial resolution of 10 meters.

Considering the high spatial resolution of Sentinel-2 images in the present study and the appropriate identification of degraded areas for mapping degradation intensity, it is suggested, based on the results of this research, to use a combination of the Albedo model and other spectral indices for monitoring and mapping degradation intensity in other dry regions as well. Given that monitoring and controlling degraded and desertified areas and preparing degradation intensity maps based on conventional models are challenging, costly, and require extensive field activities, using fast and accurate models seems highly necessary.

Furthermore, since in most studies, geomorphological units are used as working units for mapping degradation and desertification intensity, it cannot be said that the degradation intensity map obtained from these models is sufficiently accurate at small scales due to the specific land characteristics, vegetation type, and soil moisture. The development and use of such a model in the future can be a novel step toward rapidly identifying and monitoring

degraded areas in remote and inaccessible regions. Moreover, it will be effective in identifying sources of dust production and susceptibility to wind erosion.

**Declaration of interest**

The authors report no conflict of interest.

## References


1. Afrasinei, G. M., Melis, M. T., Buttau, C., Bradd, J. M., Arras, C., & Ghiglieri, G. Assessment of remote sensing-based classification methods for change detection of salt-affected areas (Biskra area, Algeria). Journal of Applied Remote Sensing. 2017; 11(1), 016025.
2. Afrasinei, G. M., Melis, M. T., Arras, C., Pistis, M., Buttau, C., & Ghiglieri, G. Spatiotemporal and spectral analysis of sand encroachment dynamics in southern Tunisia. European Journal of Remote Sensing. 2018; 51(1), 352–374.
3. Bercerril-Pina, R., Diaz-Delgado, C., Mastachi-Loza, C. A., & Gonzales-Sosa, E. Integration of remote sensing techniques for monitoring desertification in Mexico. Human and Ecological Risk Assessment. 2016; 22(6), 1323-1340.
4. Chikhaoui, M., Bonn, F., Bokoye, A, I., Merzouk, A. A spectral index for land degradation mapping using ASTER data: Application to a semi-arid Mediterranean catchment. International Journal of Applied Earth Observation and Geoformation. 2005; Vol 7. Pp 140–153. doi:10.1016/j. jag.2005.01.002.
5. Cordeiro, M. C., Santos, N. A., Alcantra Silva, V. M., Melo Luiz, D., & Silva, V. P. R. Case Study: Identification of Desertification in the years 1999, 2006 and 2011 in Mossoró-rn. Hyperspectral Remote Sensing. 2015; 5(4), 101-106.

6. Eftekhari, R., Shahriyari, A. R., & Ekhtesasi, M. R. Assessment and preparation of map for the potential and actual condition of desertification by emphasizing on wind erosion using MICD Model at the southwest of Hirmand township. Geography and Development. 2015; 13(38), 139- 150. (In Farsi).

7. Feng, J., Ding, J. L., & Wei, W. Y. A study of soil salinization in Weigan and kuqa rivers Oasis based on Albedo-MSAVI. Feature space. China Rural Water Hydropower. 2018; 2, 147–152.

8. Fozuni, L. Fakhireh, A., & Ekhtesasi, M. R. Asessmaent of desertification using of modify MEDALUS Model in Sistan plain (The east of Iran). Elixir Geoscience. 2012; 47, 8950-8955.

9. Gbetkom, Paul Gérard, Sébastien Gadal, Ahmed El Aboudi, Alfred Homère Ngandam Mfondoum, and Mamane Barkawi Mansour Badamassi. 'Mapping Change Detection of LULC on the Cameroonian Shores of Lake Chad and Its Hinterland through an Inter-Seasonal and Multisensor Approach'. International Journal of Advanced Remote Sensing and GIS. 2018; 7 (1): 2835–49.

10. Han, L., Zhang, Z., Zhang, Q., & Wan, X. Desertification assessments in the Hexi corridor of northern China's Gansu Province by remote sensing. Natural Hazards. 2015; 75(3), 2715–2731.

11. Houshyar, M. Hosseini, A. Mesgari, E. Modeling of Oroomieh Township Minimum Temperatures through Linear and Nonlinear Multiple Regression and Artificial Neural Networks Models. Geographic Notion, Zanjan University. 2012; Vol. 12. No. 82.

12. http://scihub.copernicus.eu/

13. Karnieli, A., Qin, Z., Wu, B., Panov, N., & Yan, F. Spatio-Temporal Dynamics of Land-Use and Land-Cover in the Mu Us Sandy Land, China, Using the Change Vector Analysis technique. Remote Sensing. 2014; 6(10), 9316-9339.

14. Lamchin, M., Lee, J. Y., Lee, W. K., Lee, E. J., Kim, M., Lim, C. H., Choi, H. A. & Kim, S. R. Assessment of land cover change and desertification using remote sensing technology in a local region of Mongolia. Advances in Space Research. 2016; 57(1), 64–77.

15. Li, S. G., Harazono, Y., Oikawa, T., Zhao, H. L., He, Z.Y., & Chang, X. L. Grassland desertification by grazing and the resulting micrometeorological changes in Inner Mongolia. Agricultural and Forest Meteorology. 2000; 102, 125–137.

16. Ma, Z., Xie, Y., Jiao, J., Li, L., & Wang. X. The Construction and Application of an Aledo-NDVI Based Desertification Monitoring Model. Procedia Environmental Sciences. 2011; 10(Part C), 2029 – 2035.

17. Mohammad Ghasemi, S., Zehtabian, Gh. R., & Ahmadi, H. Assessment of desertification intensity based on water index using MEDALUS methodology. Pajouhesh & Sazandgi in natural resources. 2008; 21(3-80), 59-67. (In Farsi)

18. Mzid, N.; Pignatti, S.; Huang, W.; Casa, R. An Analysis of Bare Soil Occurrence in Arable Croplands for Remote Sensing Topsoil Applications. Remote Sensing. 2021; 13, 474. https://doi.org/ 10.3390/rs13030474.

19. Naegeli, K., Damm, A., Huss, M., Wulf, H., Schaepman, M., & Hoelzle, M. Cross-Comparison of Albedo Products for Glacier Surfaces Derived from Airborne and Satellite (Sentinel-2 and Landsat 8) Optical Data. Remote Sensing. 2017; 9(2), 110.

20. Pan, J., & Li, T. Extracting desertification from Landsat TM imagery based on spectral mixture analysis and Albedo- Vegetation feature space. Natural Hazards. 2013; 68(2), 915–927.

21. Parvari Asl, S. H., Pahlavanravi, A., & Moghaddam Nia, A. R. Classification of desertification Intensity using ESAs Model in Neiyatak region (Sistan, Iran). Journal of Range and Watershed Management, Iranian Journal of Natural Resources. 2010; 63(2), 149-163. (In Farsi).

22. Polykretis, C.; Grillakis, M.G.; Alexakis, D.D. Exploring the Impact of Various Spectral Indices on Land Cover Change Detection Using Change Vector Analysis: A Case Study of Crete Island, Greece. Remote Sens. 2020; 12, 319. https://doi.org/10.3390/rs12020319.

23. Querino, C.A.S., Beneditti, C.A., Machado, N.G., da Silva, M.J.G., da Silva Querino, J.K.A., dos Santos Neto, L.A., & Biudes, M. S. Spatiotemporal NDVI, LAI, albedo, and surface temperature dynamics in the southwest of the Brazilian Amazon forest. Journal of Applied Remote Sensing. 2016; 10(2), 026007.

24. Rahimi, M., Damavandi, A. A., & Jafarian, V. Investigating remote sensing applications in evaluating and monitoring land degradation and desertification. Scientific-Research Quarterly of Geographical Data (SEPEHR). 2014; 22(88), 115-128. (In Farsi).

25. Wang, Y., Zhang, J., Tong, S., & Guo, E. Monitoring the trends of aeolian desertified lands based on time-series remote sensing data in the Horqin Sandy Land, China. Catena. 2017; 157, 286–298.

26. Wei, H., Wang, J., Cheng, K., Li, G., Ochir, A., Davaasuren, D., & Chonokhuu, S. Desertification information extraction based on feature space combinations on the Mongolian plateau. Remote Sensing. 2018; 10(10), 1614.

27. Wei, H., Wang, J., & Han, B. Desertification Information Extraction Along the China–Mongolia Railway Supported by Multisource Feature Space and Geographical Zoning Modeling. Selected Topics in Applied Earth Observations and Remote Sensing. 2020; 13, 392-402.

28. Yaduvanshi, A., Srivastava, P. K., & Pandey, A. C. Integrating TRMM and MODIS satellite with socio-economic vulnerability for monitoring drought risk over a tropical. Physics and Chemistry of the Earth, Parts A/B/C. 2015; 83(84), 14-27.

29. Zhao, Y., Wang, X., Novillo, C. J., Arrogante-Funes, P., Vázquez-Jiménez, R., & Maestre, F. T. Albedo estimated from remote sensing correlates with ecosystem multifunctionality in global drylands. Arid Environment. 2018; 157, 116–123.

30. Zolfaghari, F., Khosravi, H. Assessment of desertification severity using IMDPA model in Saravan region. Geography and Environmental Planning. 2016; 27(2-62), 87-102. (In Farsi).

31. Zolfaghari, F., Abdollahi, V. Determining the Most Suitable Vegetation Index for Mapping of Desertification Intensity in Arid Lands of Sistan Using Sentinel Images. Desert Management. 2022; Vol. 10, No. 1, Pp 1-14.

Figure 1 Click here to access/download;Figure;Fig 1.tiff

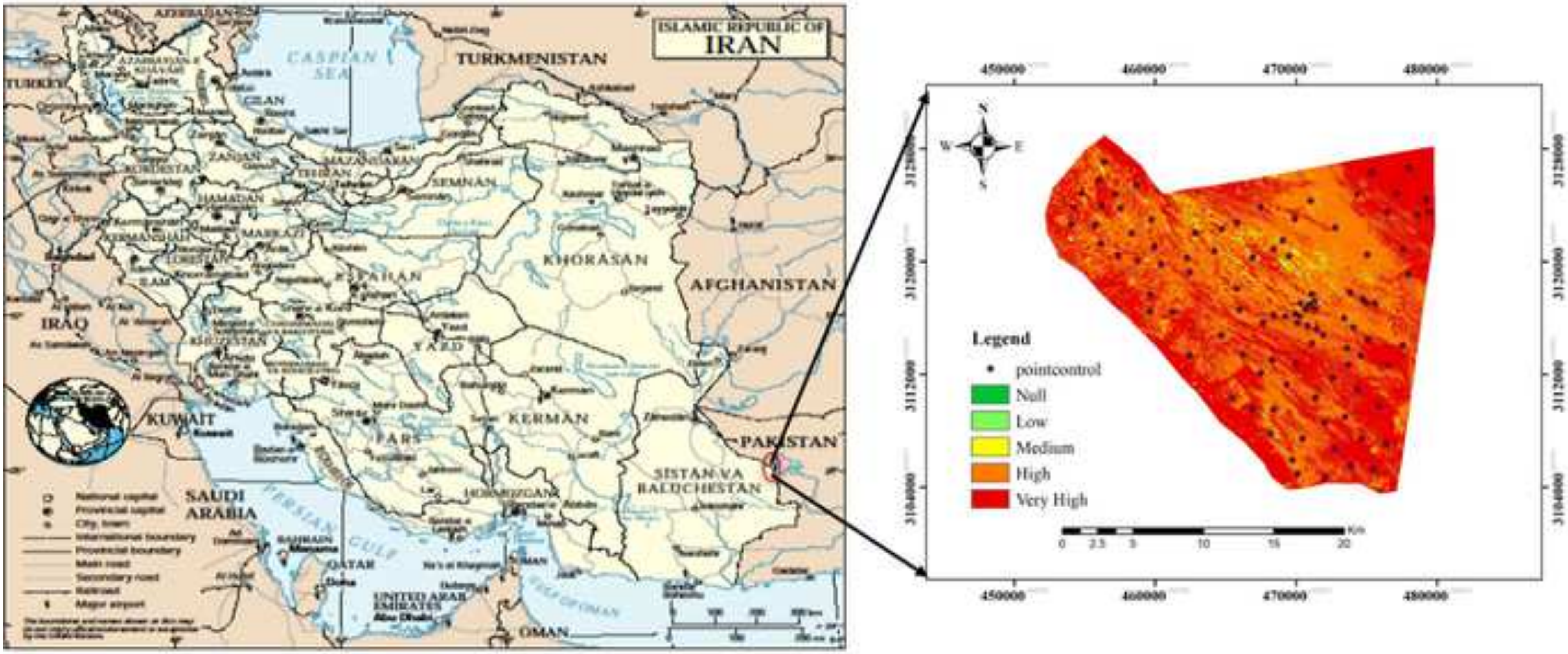

Figure 2

Click here to access/download;Figure;Fig 2.tiff

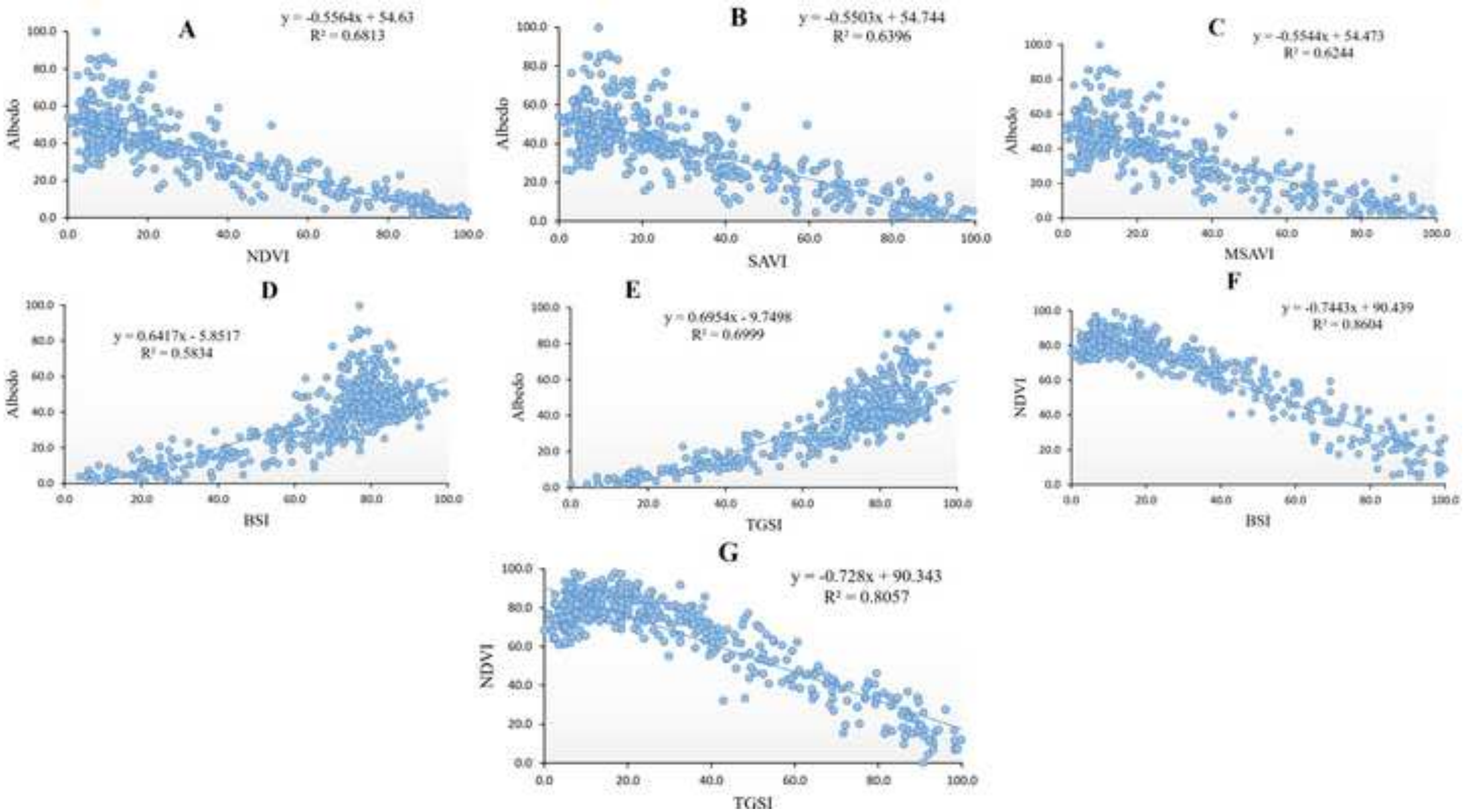

Figure 3A

Click here to access/download;Figure;Fig 3A.tiff

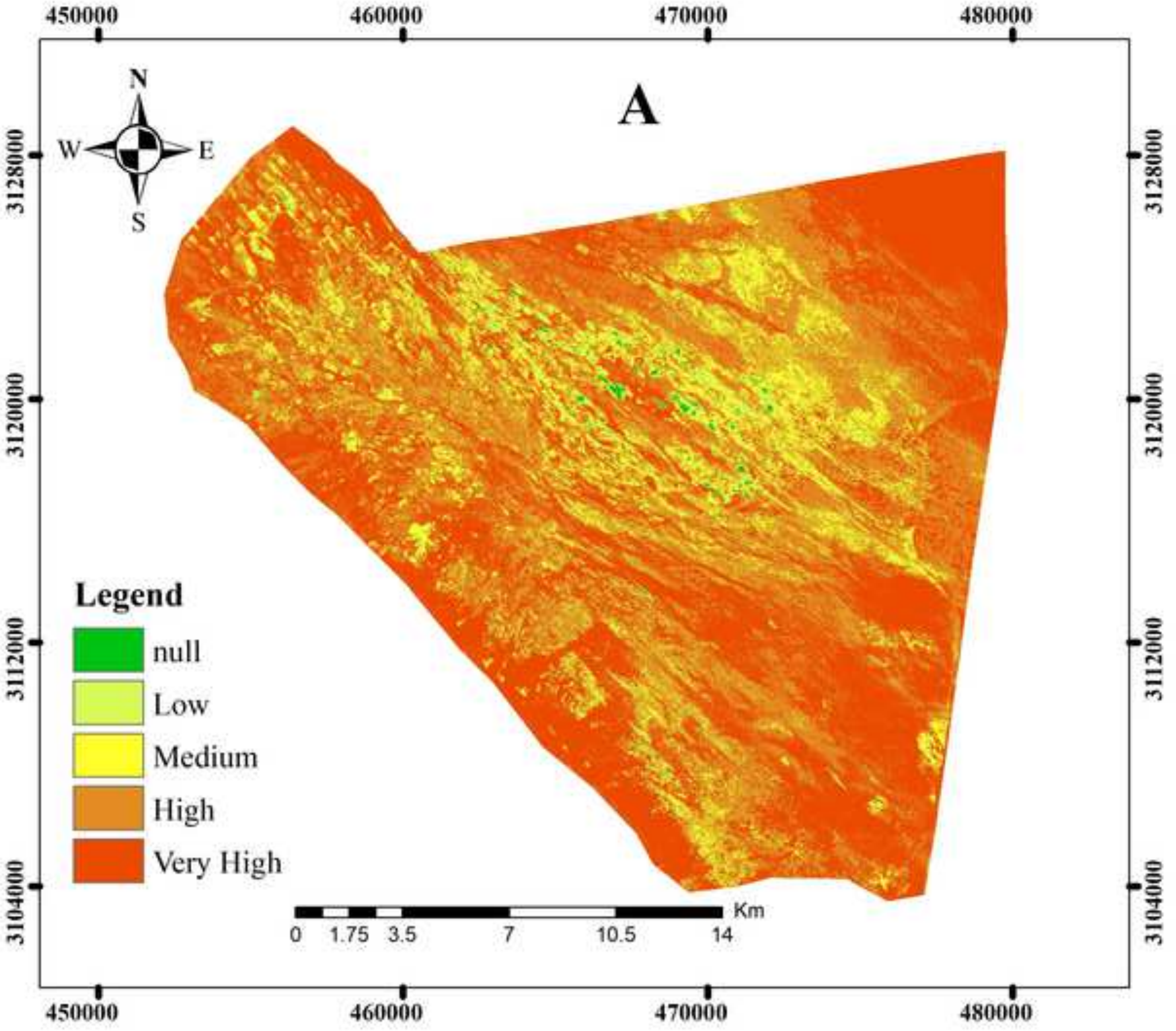

Figure 3B

Click here to access/download;Figure;Fig 3B.tiff

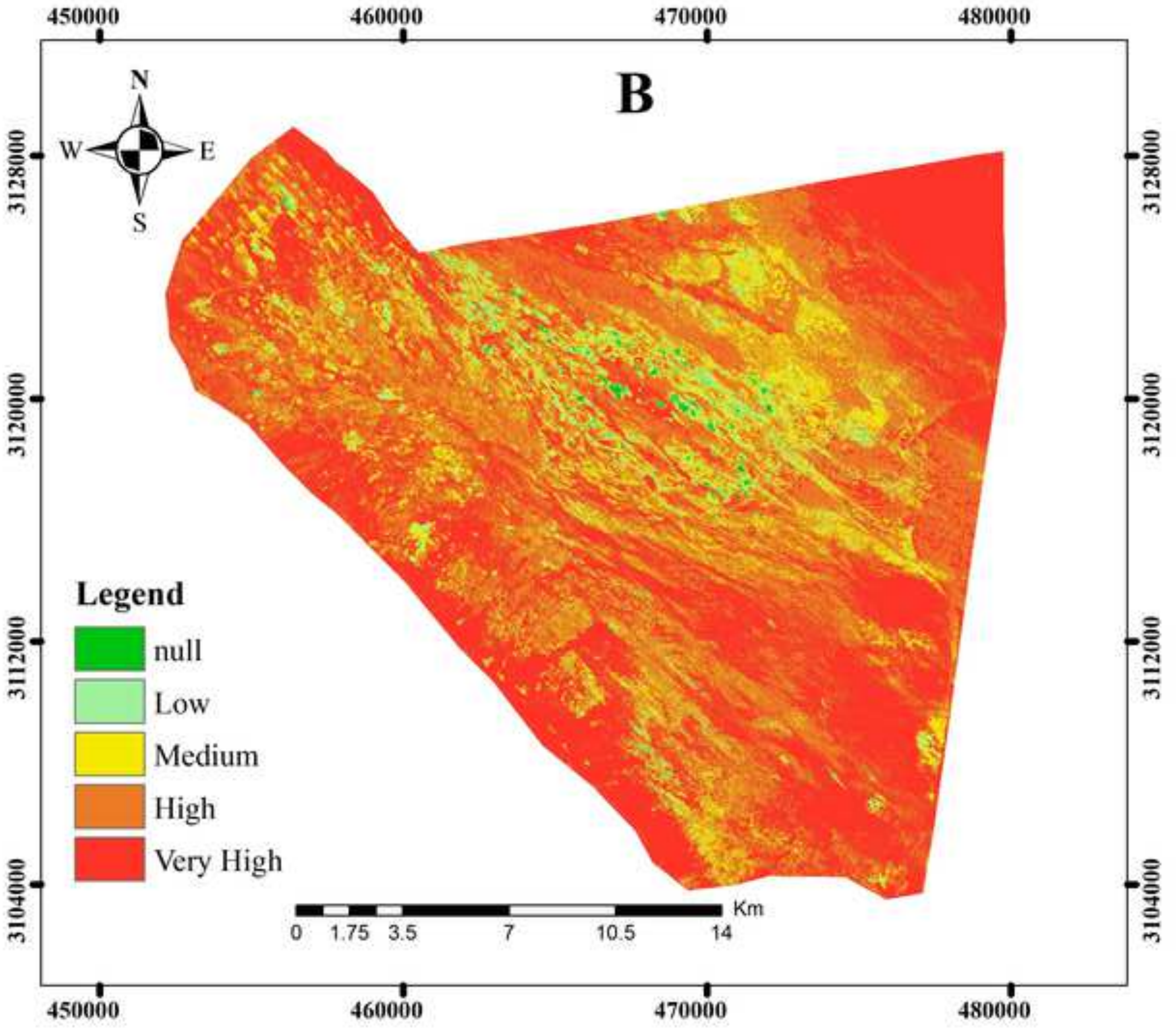

Figure 3C

Click here to access/download;Figure;Fig 3C.tiff

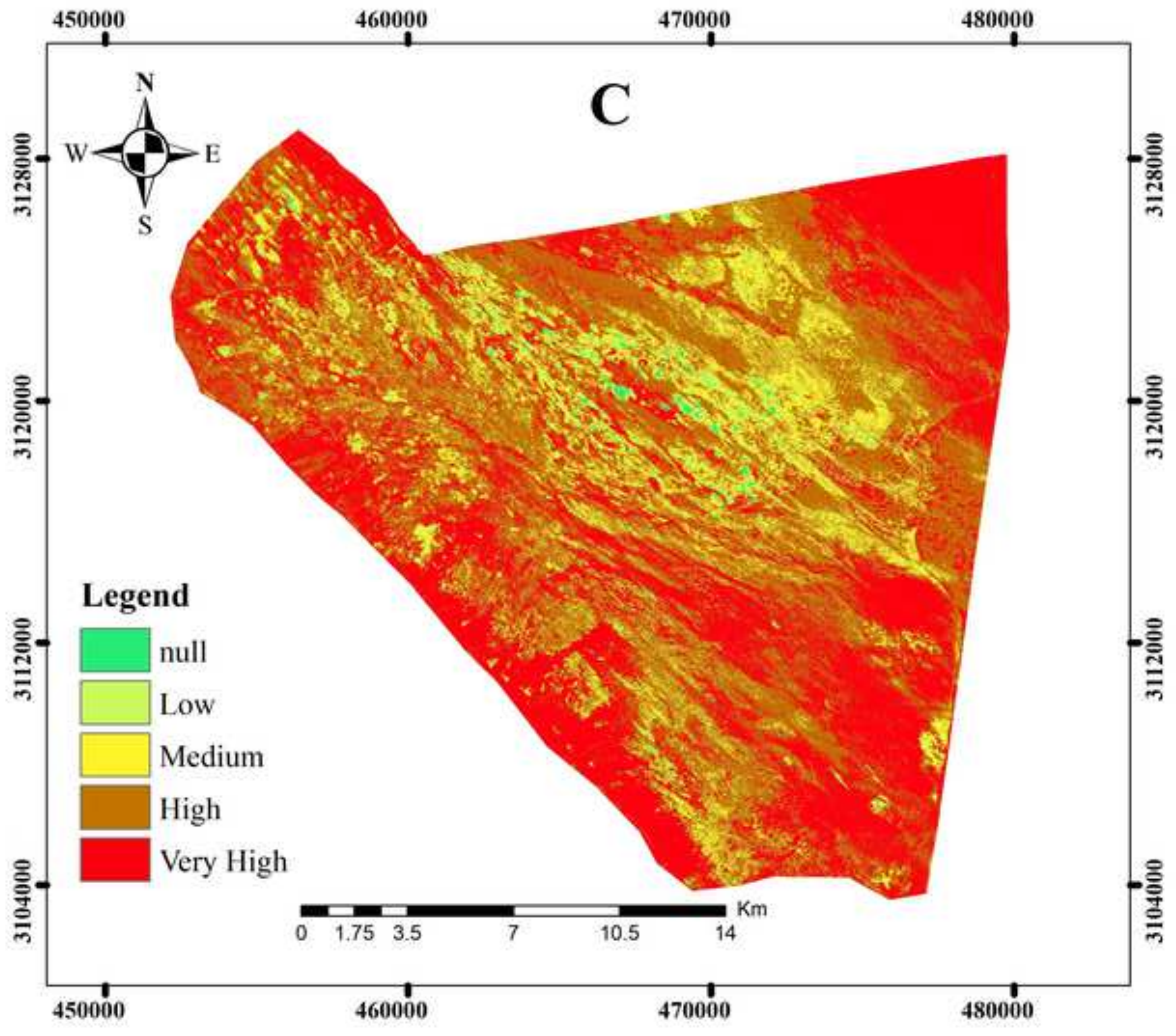

Figure 3D

Click here to access/download;Figure;Fig 3D.tiff

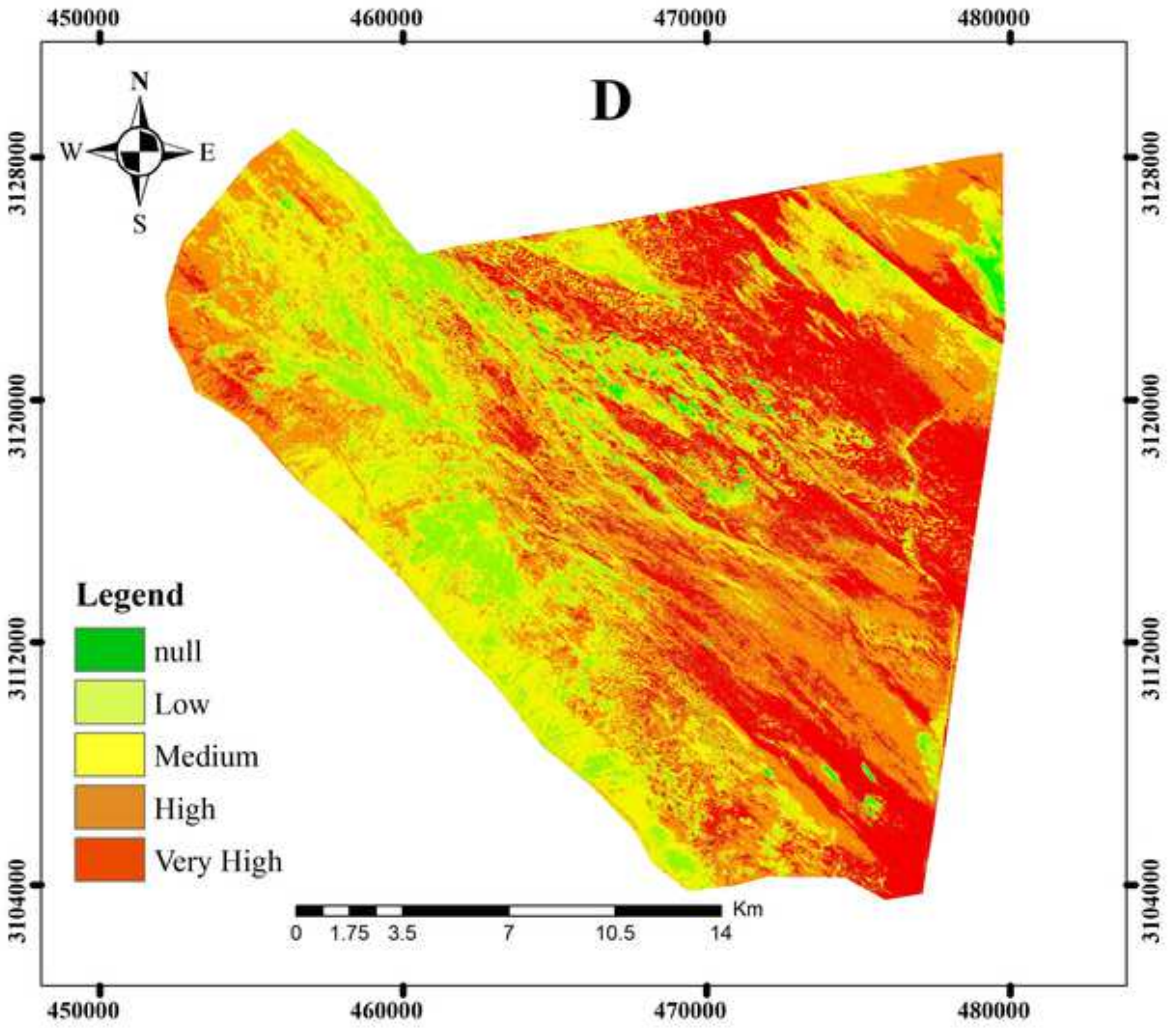

Figure 3E

Click here to access/download;Figure;Fig 3E.tiff

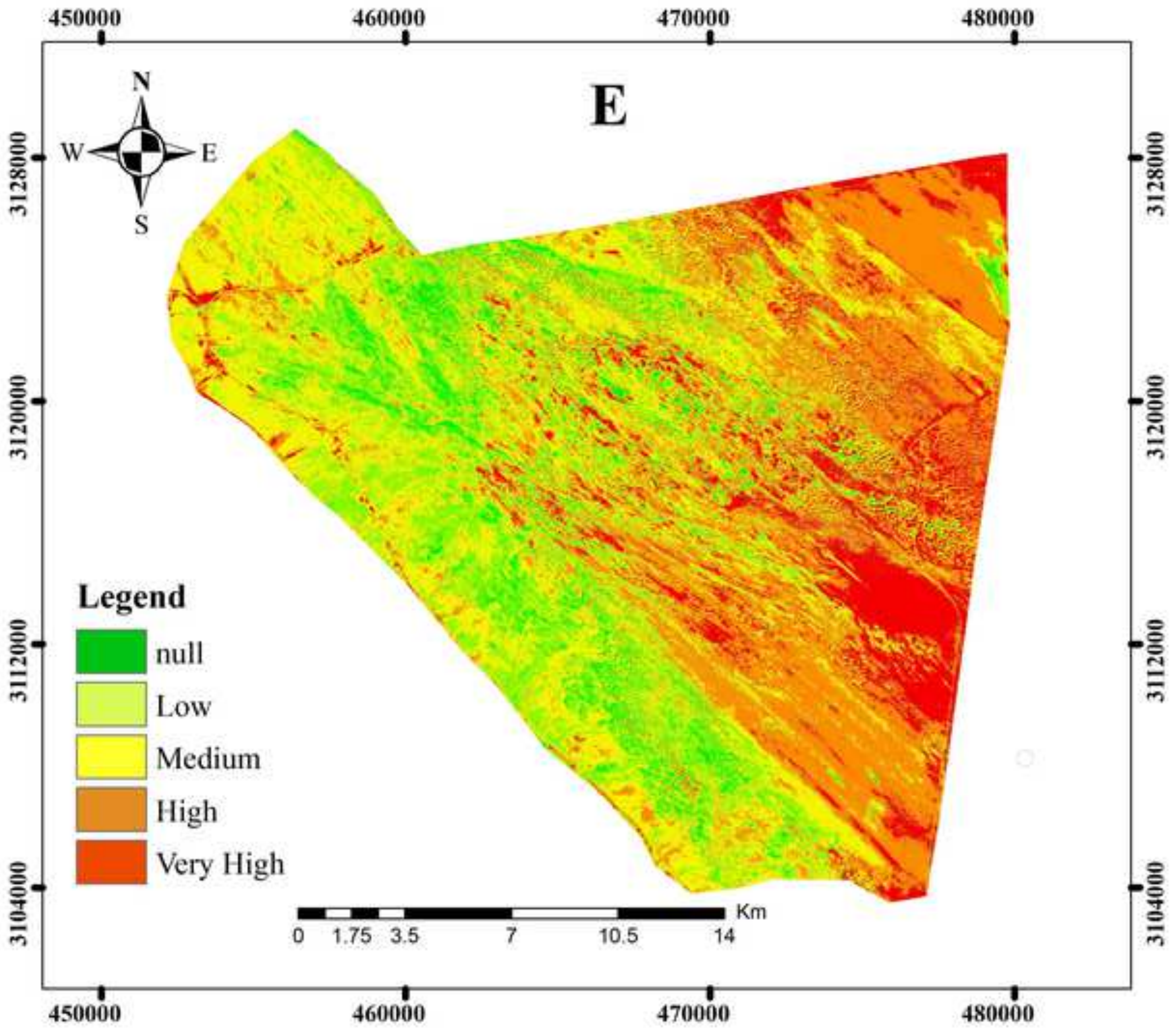

Figure 4F

Click here to access/download;Figure;Fig 4F.tiff

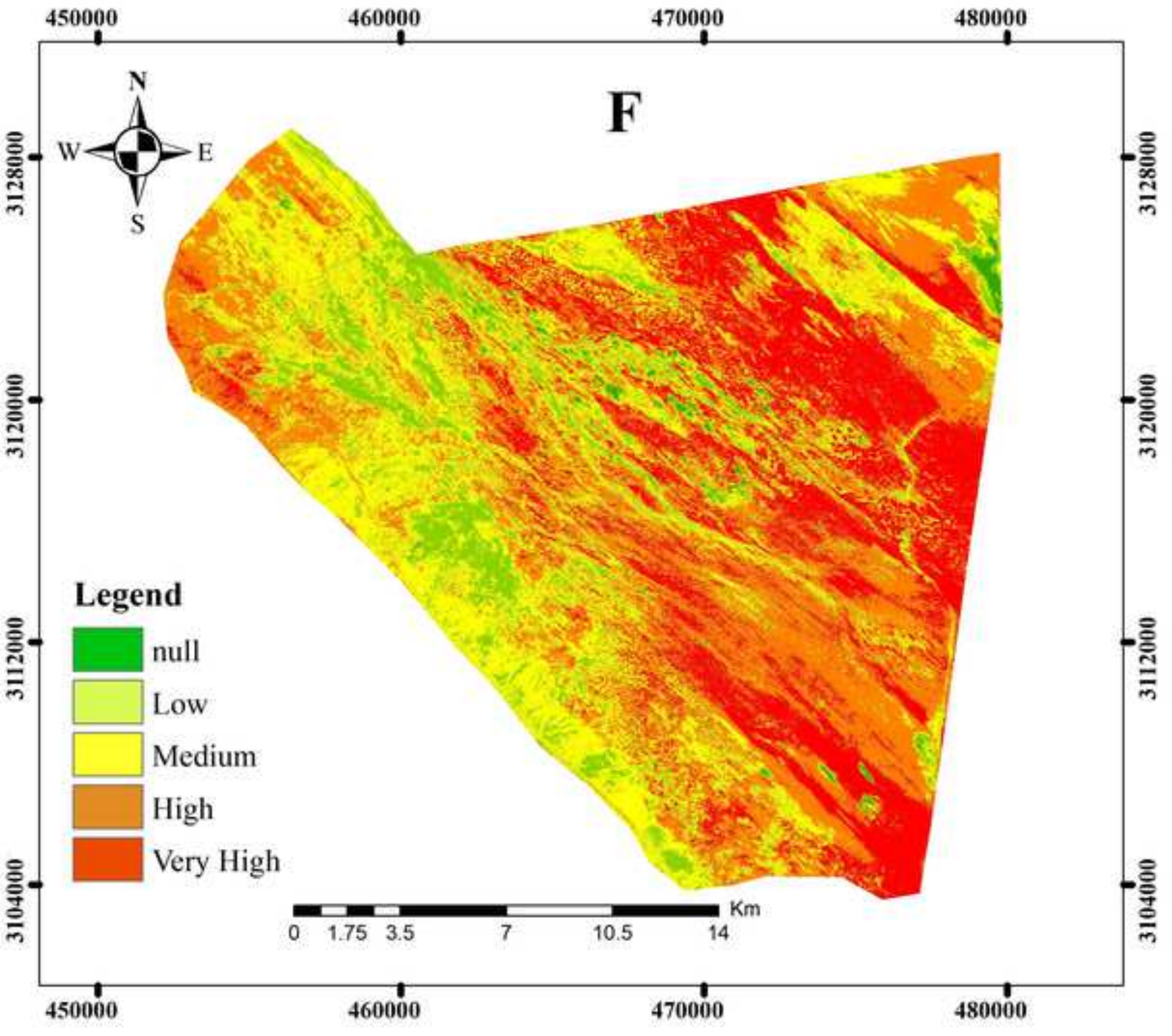

Figure 4G

Click here to access/download;Figure;Fig 4G.tiff

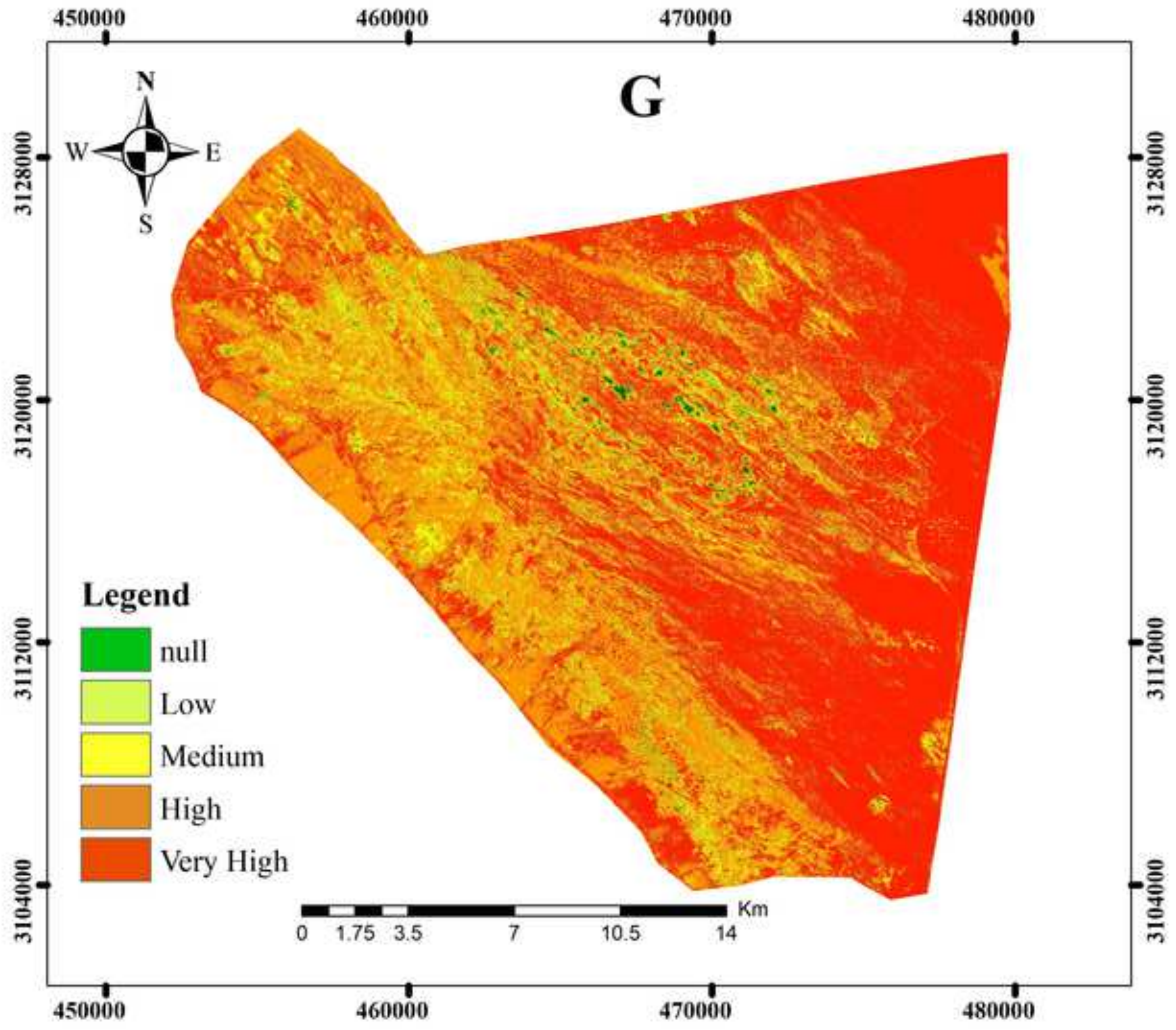

Graphycal abstract

Click here to access/download
**Supporting Information**
Graphycal abstract.pdf
