# Supplementary material for: Assessing the accuracy of spectral indices obtained from Sentinel images using field research to estimate land degradation
Source: PLoS One. 2024 Jul 25;19(7):e0305758. doi: 10.1371/journal.pone.0305758 (PMC11271892; doi:10.1371/journal.pone.0305758)
Supplement: Attachment — Submitted filename: Review PLosOne.pdf [file pone.0305758.s004.pdf]

Review PLosOne

The manuscript deals with an important subject but explanation of some points is needed.

All acronyms must be defined/described the first time they appear. Many vegetation and soil indices were used and were not defined. The proper references to such indices are also needed. Additionally, their main usage must be addressed, for instance, BSI (Bare Soil Index) is a measure of bare soil in a region and is derived by…

When describing Sentinel 2 data, please state the bands (and the region of the spectrum they cover).

Line 165 presents a brief description of the study area. This section could be improved, by adding more information about the vegetation (are annual and perennial species grassland pecies?).

Describe Equation 7 including "a" coefficient.

As it appears on the title of the manuscript, the work done in the field, at the study area, have to be described. How did you organize the data you collected in the field? Did you generate a reference map?

Please explain why you did check correlation between BSI and TGSI with NDVI (line 220). The text shows that you assume that albedo is the information used as an indicator of the absence of vegetation. What changes when you use the relations BSIxNDVI and TGSIxNDVI? Please refer to that in the discussion section.

You did not comment Table 5.

Line 295: You stated the higher accuracy (less error for consistency with field survey) was with MSAVI-albedo. Please explain why next you stated that "the NDVI-TGSI model (RMSE=0.66) outperformed other models in terms of accuracy".

Please explain what IMDPA model is.

Discussion section could show more references to similar works in other regions of the Earth.
