# Supplementary material for: Assessing the accuracy of spectral indices obtained from Sentinel images using field research to estimate land degradation
Source: PLoS One. 2024 Jul 25;19(7):e0305758. doi: 10.1371/journal.pone.0305758 (PMC11271892; doi:10.1371/journal.pone.0305758)
Supplement: Attachment — Submitted filename: Response to Reviewers .doc [file pone.0305758.s005.doc]

**Amendment Note about PONE-D-23-27523**

**" Assessing the accuracy of spectral indices obtained from Sentinel images using field research to estimate land degradation** **"**

**To Editor**

*Dear respected editor and reviewers,*

*The authors have made the necessary changes based on the reviewers’ comments and provided a point-by-point response to address the comments and revisions needed. The supporting information file “S1 Appendix” was added to answer a question posed by a referee regarding the field study. The authors note that all relevant data are within the manuscript and its supporting information files. If there are any further queries, they have requested that they be informed.*

*Best Regards,*

*Farhad Zolfaghari*

*(On behalf of all authors)*

**To academic editor**

1- Manuscript style requirements were checked.

2- The implementation of the field project did not require a permit due to the region’s governing laws, and public access to the area is free.

3- the statement “The funders had no role in study design, data collection and analysis, decision to publish, or preparation of the manuscript. The authors received no specific funding for this work“, were added to “Declaration of interest“ section.

4-You have raised an important point in relation with images which may be copyrighted. Figure 1 in the manuscript was downloaded from NASA Earth Observatory (public domain):<http://earthobservatory.nasa.gov/> (public domain).

The sentinel data required for figures 3 and 4 was obtained from the Open Access Hub of the European Space Agency, which is publicly accessible at https://scihub.copernicus.eu/dhus/#/home 1. After extracting the analyzed indicators in Snap and ArcGIS software, figures 3 and 4 were produced, which represent the output of the software

5- Many thanks for pointing this out. I have revised it in text.

6- Many thanks for pointing this out. I have revised it in text.

7- Many thanks for pointing this out. I have revised it in text.

**To Reviewer #1**

1. References cited in the text are not as per PLOSONE Journal format.

**Reply:** Agreed, as I have revised it in text.

2. Expand the abbreviations of indices at the time of first occurrence.

**Reply:** Many thanks for pointing this out. I have revised it in text.

3. Line 96, correct the word sentinel as sentinel2.

**Reply:** Thank you for your thorough review and salient observation. I have revised it in text.

4. Several studies addressed the issue of land degradation and desertification with the use of Albedo, NDVI and TGSI (Ma et al., 2011; Chen et al., 2021; Bazgeer et al., 2019; Aramesh et al., 2022; Lamqadem et al., 2018) using sentine2 and other satellite datasets. Describe the goals and objectives of the study with innovation and how it differs with other studies.

**Reply:** Many thanks for pointing this out. Validation of different spectral indices and field studies to determine the best spectral index for accurately assessing land degradation. This validation is essential due to the characteristics of the type of cover and the unique conditions of areas affected by the destruction. this process requires a scientific approach to identify the factors that contribute to land degradation and determine their intensity. The validation process involves a rigorous evaluation of the factors that contribute to degradation, including soil, vegetation, wind erosion, and climate criteria. this validation process is crucial because it helps to ensure that the best spectral index is used to accurately assess land degradation. The explanation has been added to the introduction section, which provides an overview of the research topic and highlights its significance. (Pages: 4 and 5, Lines: 98-109)

5. The authors described in the Line, 182, that the accuracy of the study was tested with 100 ground control points and field observations. However, the details on ground data collection, parameters used, use of any portable or suitable devices for the measurement of albedo, and other tested vegetation indexes are not clear.

**Reply:** Thank you for your thorough review and salient observation. for example, for plat 1, the climate indicator was calculated based on the geometric mean of the weights of three indices including precipitation, drought index, and drought event:

Moreover, the degradation severity of the plat 1 was calculated using the geometric mean weight of four relevant indicators (climate, soil, wind erosion, and vegetation):

Then, according to the table below, the degradation intensity of each plot was determined.

| Class of desertification | Range of score |
| --- | --- |
| Low | 1-1.24 |
| Medium | 1.25-1.49 |
| High | 1.5-1.74 |
| Very High | 1.75-2 |

The full description of the field study is added in Supporting Information files as “S1 Apendix”

**To Reviewer #2**

- This manuscript does not contain sufficient novelty . . .

**Reply:** The reviewer raises an interesting concern. However, we feel the reviewer may not fully comprehend the scope of the work. This study is designed to address the question of how different spectral indices extracted from satellite images can be utilized to identify areas affected by destruction. It is important to note that the applicability of these indices may vary across different geographical regions. To achieve this objective, the relationship between the albedo index and indicators associated with bare soil surface and vegetation was examined to quantify the extent of destruction. Additionally, a field-based approach was employed, involving the investigation of 100 plots, to determine which indicators yield the most accurate results in assessing the intensity of destruction. These plots were used to evaluate criteria related to soil, vegetation, wind erosion, and climate within the study area. Finally, the Mann-Whitney statistical model was employed to identify and introduce the best model derived from spectral indices

**To Reviewer #3**

1. All acronyms must be defined/described the first time they appear.

**Reply:** Many thanks for pointing this out. I have revised it in text.

2. Many vegetation and soil indices were used and were not defined. The proper references to such indices are also needed. Additionally, their main usage must be addressed, for instance, BSI (Bare Soil Index) is a measure of bare soil in a region and is derived by…

**Reply:** Thank you for bringing this to our attention. We have added the explanation and Table 2 to the methodology section, as per your suggestion. The relevant pages are 7-9, and the lines are 169-188.

3. When describing Sentinel 2 data, please state the bands (and the region of the spectrum they cover).

**Reply:** Thank you for your thorough review and salient observation. The explanation and Table 1 have been added to the document (Page: 7, Lines: 155-167).

4. Line 165 presents a brief description of the study area. This section could be improved, by adding more information about the vegetation (are annual and perennial species grassland pecies?).

**Reply:** Thank you for your constructive comments and suggestions. The vegetation in the area is predominantly comprised of bush and shrub species, which are sparsely distributed in the flood plains. Due to inadequate precipitation, several areas of the vegetation cover have desiccated. Herbaceous and annual species have nearly vanished, leading the land that was previously utilized as pasture for light livestock to transform into completely arid regions with scanty bush cover. Table 3 presents the floristic list of the study area. The materials and methods section has been updated with this information. (Page: 6, Lines: 130-134).

Floristic list of the study area

| **ردیف** | **نام علمی گونه** | **ردیف** | **نام علمی گونه** |
| --- | --- | --- | --- |
| ***1*** | *Tamarix aphylla* | 11 | *Anabasis steifira* |
| ***2*** | *Hamada salicornica* | 12 | *Atriplex leucoclad* |
| ***3*** | *Haloxylon persicum* | 13 | *Cusinia gadrosisa* |
| ***4*** | *Caparis spinosa* | 14 | *Echinops sedrosiaca* |
| ***5*** | *Aellenia ariculata* | 15 | *Allysum dasycarpum* |
| ***6*** | *Aellenia subaph* | 16 | *Citrullus colocynthis* |
| ***7*** | *Agriophyllum minus* | 17 | *Carex physodes* |
| ***8*** | *Peganum harmala* | 18 | *Bromus tectorum* |
| ***9*** | *Cynodon dactylon* | 19 | *Calligonum bungei* |
| ***10*** | *Cousinia stocksii* | 20 | *Nitraria schoberi* |

5. Describe Equation 7 including “a” coefficient.

**Reply:** Many thanks for pointing this out. The (a) value is the slope of the orthogonal lines found in the NDVI, SAVI, MSAVI, BSI, and TGSI separately with Albedo relationship, as added in the text.

6. As it appears on the title of the manuscript, the work done in the field, at the study area, have to be described. How did you organize the data you collected in the field? Did you generate a reference map?

**Reply:** Thank you for your constructive comments. In our field operations, we randomly selected 100 plots of 10 x 10 square meters in different parts of the study area. For each plot, we evaluated four criteria: vegetation cover, wind erosion, climate, and soil quality. We used indicators defined for each criterion based on an Iranian Model of Desertification Potential Assessment (IMDPA) model. The summary of the field model we investigated is presented in S1 Appendix due to the article’s increased volume.

7. Please explain why you did check correlation between BSI and TGSI with NDVI (line 220).

**Reply:** Thank you for your constructive feedback. We believe that it would be beneficial to examine and investigate the correlation between various indices to identify the most effective spectral indices that can indicate the severity of degradation. As stated in the introduction, this research aims to address the question of identifying the optimal spectral index for determining degradation intensity. During our evaluation of various indicators with field data, we were intrigued by how we could assess the results of these indicators with field data. As revised in the text (page: 15, lines 335-347)

8. The text shows that you assume that albedo is the information used as an indicator of the absence of vegetation. What changes when you use the relations BSIxNDVI and TGSIxNDVI? Please refer to that in the discussion section.

**Reply:** The reviewer raises an interesting concern. I have revised it and have added to the text (Page: 19, Line: 428-440).

9. You did not comment Table 5.

**Reply:** Agree. Many thanks for pointing this out. I have revised it in text.

10. Line 295: You stated the higher accuracy (less error for consistency with field survey) was with MSAVI-albedo. Please explain why next you stated that “the NDVI-TGSI model (RMSE=0.66) outperformed other models in terms of accuracy”.

**Reply:** Many thanks for pointing this out. I have revised it (Page: 15, Lines: 335-347). We think these changes are now better. We hope that you agree.

11. Please explain what IMDPA model is.

**Reply:** The explanation of the Iranian Model Desertification Potential Assessment (IMDPA) has been added to the S1 Appendix supporting file.

12. Discussion section could show more references to similar works in other regions of the Earth.

**Reply:** Many thanks for pointing this out. as I have revised it in text (Page: 18, Lines: 407-415).
