# Supplementary material for: Assessing the accuracy of spectral indices obtained from Sentinel images using field research to estimate land degradation
Source: PLoS One. 2024 Jul 25;19(7):e0305758. doi: 10.1371/journal.pone.0305758 (PMC11271892; doi:10.1371/journal.pone.0305758)
Supplement: Attachment — Submitted filename: Response to Reviewers .doc [file pone.0305758.s006.doc]

**Amendment Note about PONE-D-23-27523**

**" Assessing the accuracy of spectral indices obtained from Sentinel images using field research to estimate land degradation** **"**

**To Editor**

*Dear respected editor and reviewers,*

*The authors have made the necessary changes based on the reviewers’ comments and provided a point-by-point response to address the comments and revisions needed. An additional reference has been incorporated into the Methods section (page 9, line 196). This new source is now listed as reference number 39 in the references section (page 26, line 571).*

*The authors note that all relevant data are within the manuscript and its supporting information files. If there are any further queries, they have requested that they be informed.*

*Best Regards,*

*Farhad Zolfaghari*

*(On behalf of all authors)*

**To Reviewer #1**

1. Authors attempted to enhance the quality of the manuscript. However, minor modifications as follows: 1. Line 99: Expand the abbreviation "IMDPA", as it is first occurrence, in the manuscript. Provide a reference.

**Reply:** Agreed, as I have revised it in text. (Page: 4, Lines: 99-100)

2. Table 1: spectral bands of MSI sensor - As authors used onlt 10 m bands of sentinel image. This information is available at sentinel-2 website; hence the reference is enough. It is recommended to remove.

**Reply:** Many thanks for pointing this out. I have revised it in text. (Page: 6, Line: 146)

3. Table 5: Provide scale of values to severity degradation class. (for example: low ??, describe the range in scale)

**Reply:** Thank you for your thorough review and salient observation. I have revised it in text. (Page: 13, Line: 300).

4. The description provided in Appendix-S1 is a key for field data and its analysis. It should be in the methods section of the main/manuscript. However, the tables can be provided in S1 Appendix.

**Reply:** Thank you for your thorough review and salient observation. The description of the field study is added in methodology section. (Pages: 9 and 10, Lines: 199-219)
